# Supplementary material for: Identification of a biological form in the Anopheles stephensi laboratory colony using the odorant-binding protein 1 intron I sequence
Source: PLoS One. 2022 Feb 22;17(2):e0263836. doi: 10.1371/journal.pone.0263836 (PMC8863247; doi:10.1371/journal.pone.0263836)
Supplement: S1 Fig — Bootstrap values >70 shown at nodes. (DOCX) [file pone.0263836.s001.docx]

#MW012492 TCA CAC ATT ATT ACT CAA GAA AGA GGT AAA AAG GAA ACA TTC GGA AAT TTA GGA ATA ATT [ 60]

#MZ269698 ... ... ... ... ... ... ... ... ... ... ... ... ... ... ... ... ... ... ... ... [ 60]

#MZ269699 ... ... ... ... ... ... ... ... ... ... ... ... ... ... ... ... ... ... ... ... [ 60]

#MZ269700 ... ... ... ... ... ... ... ... ... ... ... ... ... ... ... ... ... ... ... ... [ 60]

#MZ269701 ... ... ... ... ... ... ... ... ... ... ... ... ... ... ... ... ... ... ... ... [ 60]

#MZ269702 ... ... ... ... ... ... ... ... ... ... ... ... ... ... ... ... ... ... ... ... [ 60]

#MZ269703 ... ... ... ... ... ... ... ... ... ... ... ... ... ... ... ... ... ... ... ... [ 60]

#MZ269704 ... ... ... ... ... ... ... ... ... ... ... ... ... ... ... ... ... ... ... ... [ 60]

#MZ269705 ... ... ... ... ... ... ... ... ... ... ... ... ... ... ... ... ... ... ... ... [ 60]

#KT899888 ... ... ... ... ... ... ... ... ... ... ... ... ... ... ... ... ... ... ... ... [ 60]

#AY877426 ... ... ... ... ... ... ... ... ... ... ... ... ... ... ... ... ... ... ... ... [ 60]

#AY877428 ... ... ... ... ... ... ... ... ... ... ... ... ... ... ... ... ... ... ... ... [ 60]

#AY877429 ... ... ... ... ... ... ... ... ... ... ... ... ... ... ... ... ... ... ... ... [ 60]

#AY877427 ... ... ... ... ... ... ... ... ... ... ... ... ... ... ... ... ... ... ... ... [ 60]

#AF417713 ... ... ... ... ... ... ... ... ... ... ... ... ... ... ... ... ... ... ... ... [ 60]

#KR817728 ... ... ... ... ... ... ... ... ... ... ... ... ... ... ... ... ... ... ... ... [ 60]

#MW012492 TAT GCT ATA TTA GCA ATT GGA TTA CTT GGA TTT ATC GTA TGA GCC CAC CAT ATG TTT ACA [120]

#MZ269698 ... ... ... ... ... ... ... ... ... ... ... ... ... ... ... ... ... ... ... ... [120]

#MZ269699 ... ... ... ... ... ... ... ... ... ... ... ... ... ... ... ... ... ... ... ... [120]

#MZ269700 ... ... ... ... ... ... ... ... ... ... ... ... ... ... ... ... ... ... ... ... [120]

#MZ269701 ... ... ... ... ... ... ... ... ... ... ... ... ... ... ... ... ... ... ... ... [120]

#MZ269702 ... ... ... ... ... ... ... ... ... ... ... ... ... ... ... ... ... ... ... ... [120]

#MZ269703 ... ... ... ... ... ... ... ... ... ... ... ... ... ... ... ... ... ... ... ... [120]

#MZ269704 ... ... ... ... ... ... ... ... ... ... ... ... ... ... ... ... ... ... ... ... [120]

#MZ269705 ... ... ... ... ... ... ... ... ... ... ... ... ... ... ... ... ... ... ... ... [120]

#KT899888 ... ... ... ... ... ... ... ... ... ... ... ... ... ... ... ... ... ... ... ... [120]

#AY877426 ... ... ... ... ... ... ... ... ... ... ... ..T ... ... ... ... ... ... ... ... [120]

#AY877428 ... ... ... ... ... ... ... ... ... ... ... ..T ... ... ... ... ... ... ... ... [120]

#AY877429 ... ... ... ... ... ... ... ... ... ... ... ..T ... ... ... ... ... ... ... ... [120]

#AY877427 ... ... ... ... ... ... ... ... ... ... ... ..T ... ... ... ... ... ... ... ... [120]

#AF417713 ... ... ... ... ... ... ... ... ... ... ... ... ... ... ... ... ... ... ... ... [120]

#KR817728 ... ... ... ... ... ... ... ... ... ... ... ..T ... ... ... ... ... ... ... ... [120]

#MW012492 GTA GGA ATA GAC GTA GAT ACT CGA GCT TAT TTT ACA TCA GCT ACA ATA ATT ATT GCT GTT [180]

#MZ269698 ... ... ... ... ... ... ... ... ... ... ... ... ... ... ... ... ... ... ... ... [180]

#MZ269699 ... ... ... ... ... ... ... ... ... ... ... ... ... ... ... ... ... ... ... ... [180]

#MZ269700 ... ... ... ... ... ... ... ... ... ... ... ... ... ... ... ... ... ... ... ... [180]

#MZ269701 ... ... ... ... ... ... ... ... ... ... ... ... ... ... ... ... ... ... ... ... [180]

#MZ269702 ... ... ... ... ... ... ... ... ... ... ... ... ... ... ... ... ... ... ... ... [180]

#MZ269703 ... ... ... ... ... ... ... ... ... ... ... ... ... ... ... ... ... ... ... ... [180]

#MZ269704 ... ... ... ... ... ... ... ... ... ... ... ... ... ... ... ... ... ... ... ... [180]

#MZ269705 ... ... ... ... ... ... ... ... ... ... ... ... ... ... ... ... ... ... ... ... [180]

#KT899888 ... ... ... ... ... ... ... ... ... ... ... ... ... ... ... ... ... ... ... ... [180]

#AY877426 ... ... ... ... ... ... ... ... ... ... ... ... ... ... ... ... ... ... ... ... [180]

#AY877428 ... ... ... ... ... ... ... ... ... ... ... ... ... ... ... ... ... ... ... ... [180]

#AY877429 ... ... ... ... ... ... ... ... ... ... ... ... ... ... ... ... ... ... ... ... [180]

#AY877427 ... ... ... ... ... ... ... ... ... ... ... ... ... ... ... ... ... ... ... ... [180]

#AF417713 ... ... ... ... ... ... ... ... ... ... ... ... ... ... ... ... ... ... ... ... [180]

#KR817728 ... ... ... ... ... ... ... ... ... ... ... ... ... ... ... ... ... ... ... ... [180]

#MW012492 CCA ACT GGA ATT AAA ATT TTT AGT TGA TTA GCA ACA TTA CAC GGA ACA CAA CTT ACT TAT [240]

#MZ269698 ... ... ... ... ... ... ... ... ... ... ... ... ... ... ... ... ... ... ... ... [240]

#MZ269699 ... ... ... ... ... ... ... ... ... ... ... ... ... ... ... ... ... ... ... ... [240]

#MZ269700 ... ... ... ... ... ... ... ... ... ... ... ... ... ... ... ... ... ... ... ... [240]

#MZ269701 ... ... ... ... ... ... ... ... ... ... ... ... ... ... ... ... ... ... ... ... [240]

#MZ269702 ... ... ... ... ... ... ... ... ... ... ... ... ... ... ... ... ... ... ... ... [240]

#MZ269703 ... ... ... ... ... ... ... ... ... ... ... ... ... ... ... ... ... ... ... ... [240]

#MZ269704 ... ... ... ... ... ... ... ... ... ... ... ... ... ... ... ... ... ... ... ... [240]

#MZ269705 ... ... ... ... ... ... ... ... ... ... ... ... ... ... ... ... ... ... ... ... [240]

#KT899888 ... ... ... ... ... ... ... ... ... ... ... ... ... ... ... ... ... ... ... ... [240]

#AY877426 ... ... ... ... ... ... ... ... ... ... ..T ... ... ... ... ... ... ... ... ... [240]

#AY877428 ... ... ... ... ... ... ... ... ... ... ..T ... ... ... ... ... ... ... ... ... [240]

#AY877429 ... ... ... ... ... ... ... ... ... ... ..T ... ... ... ... ... ... ... ... ... [240]

#AY877427 ... ... ... ... ... ... ... ... ... ... ..T ... ... ... ... ... ... ... ... ... [240]

#AF417713 ... ... ... ... ... ... ... ... ... ... ..T ... ... ... ... ... ... ... ... ... [240]

#KR817728 ... ... ... ... ... ... ... ... ... ... ..T ... ... ... ... ... ... ... ... ... [240]

#MW012492 AGC CCA GCT ATA TTA TGA GCA TTT GGA TTT GTA TTT TTA TTT ACA GTT GGA GGA TTA ACT [300]

#MZ269698 ... ... ... ... ... ... ... ... ... ... ... ... ... ... ... ... ... ... ... ... [300]

#MZ269699 ... ... ... ... ... ... ... ... ... ... ... ... ... ... ... ... ... ... ... ... [300]

#MZ269700 ... ... ... ... ... ... ... ... ... ... ... ... ... ... ... ... ... ... ... ... [300]

#MZ269701 ... ... ... ... ... ... ... ... ... ... ... ... ... ... ... ... ... ... ... ... [300]

#MZ269702 ... ... ... ... ... ... ... ... ... ... ... ... ... ... ... ... ... ... ... ... [300]

#MZ269703 ... ... ... ... ... ... ... ... ... ... ... ... ... ... ... ... ... ... ... ... [300]

#MZ269704 ... ... ... ... ... ... ... ... ... ... ... ... ... ... ... ... ... ... ... ... [300]

#MZ269705 ... ... ... ... ... ... ... ... ... ... ... ... ... ... ... ... ... ... ... ... [300]

#KT899888 ... ... ... ... ... ... ... ... ... ... ... ... ... ... ... ... ... ... ... ... [300]

#AY877426 ... ... ... ... ... ... ... ... ... ... ... ... ... ... ... ... ... ... ... ... [300]

#AY877428 ... ... ... ... ... ... ... ... ... ... ... ... ... ... ... ... ... ... ... ... [300]

#AY877429 ... ... ... ... ... ... ... ... ... ... ... ... ... ... ... ... ... ... ... ... [300]

#AY877427 ... ... ... ... ... ... ... ... ... ... ... ... ... ... ... ... ... ... ... ... [300]

#AF417713 ... ... ... ... ... ... ... ... ... ... ... ... ... ... ... ... ... ... ... ... [300]

#KR817728 ... ... ... ... ... ... ... ... ... ... ... ... ... ... ... ... ... ... ... ... [300]

#MW012492 GGA GTT GTT TTA GCA AAT TCA TCA ATT GAT ATT GTT TTA CAT GAT ACT TAT TAT GTT GTA [360]

#MZ269698 ... ... ... ... ... ... ... ... ... ... ... ... ... ... ... ... ... ... ... ... [360]

#MZ269699 ... ... ... ... ... ... ... ... ... ... ... ... ... ... ... ... ... ... ... ... [360]

#MZ269700 ... ... ... ... ... ... ... ... ... ... ... ... ... ... ... ... ... ... ... ... [360]

#MZ269701 ... ... ... ... ... ... ... ... ... ... ... ... ... ... ... ... ... ... ... ... [360]

#MZ269702 ... ... ... ... ... ... ... ... ... ... ... ... ... ... ... ... ... ... ... ... [360]

#MZ269703 ... ... ... ... ... ... ... ... ... ... ... ... ... ... ... ... ... ... ... ... [360]

#MZ269704 ... ... ... ... ... ... ... ... ... ... ... ... ... ... ... ... ... ... ... ... [360]

#MZ269705 ... ... ... ... ... ... ... ... ... ... ... ... ... ... ... ... ... ... ... ... [360]

#KT899888 ... ... ... ... ... ... ... ... ... ... ... ... ... ... ... ... ... ... ... ... [360]

#AY877426 ... ... ... ... ... ... ... ... ... ... ... ... ... ... ... ... ... ... ... ... [360]

#AY877428 ... ... ... ... ... ... ... ... ... ... ... ... ... ... ... ... ... ... ... ... [360]

#AY877429 ... ... ... ... ... ... ... ... ... ... ... ... ... ... ... ... ... ... ... ... [360]

#AY877427 ... ... ... ... ... ... ... ... ... ... ... ... ... ... ... ... ... ... ... ... [360]

#AF417713 ... ... ... ... ... ... ... ... ... ... ... ... ... ... ... ... ... ... ... ... [360]

#KR817728 ... ... ... ... ... ... ... ... ... ... ... ... ... ... ... ... ... ... ... ... [360]

#MW012492 GCT CAT TTT CAT TAT GTC TTA TCA ATA GGA GCA GTA TTT GCT ATT ATA GCA GGA TTT ATT [420]

#MZ269698 ... ... ... ... ... ... ... ... ... ... ... ... ... ... ... ... ... ... ... ... [420]

#MZ269699 ... ... ... ... ... ... ... ... ... ... ... ... ... ... ... ... ... ... ... ... [420]

#MZ269700 ... ... ... ... ... ... ... ... ... ... ... ... ... ... ... ... ... ... ... ... [420]

#MZ269701 ... ... ... ... ... ... ... ... ... ... ... ... ... ... ... ... ... ... ... ... [420]

#MZ269702 ... ... ... ... ... ... ... ... ... ... ... ... ... ... ... ... ... ... ... ... [420]

#MZ269703 ... ... ... ... ... ... ... ... ... ... ... ... ... ... ... ... ... ... ... ... [420]

#MZ269704 ... ... ... ... ... ... ... ... ... ... ... ... ... ... ... ... ... ... ... ... [420]

#MZ269705 ... ... ... ... ... ... ... ... ... ... ... ... ... ... ... ... ... ... ... ... [420]

#KT899888 ... ... ... ... ... ... ... ... ... ... ... ... ... ... ... ... ... ... ... ... [420]

#AY877426 ... ... ... ... ... ... ... ... ... ... ... ... ... ... ... ... ... ... ... ... [420]

#AY877428 ... ... ... ... ... ... ... ... ... ... ... ... ... ... ... ... ... ... ... ... [420]

#AY877429 ... ... ... ... ... ... ... ... ... ... ... ... ... ... ... ... ... ... ... ... [420]

#AY877427 ... ... ... ... ... ... ... ... ... ... ... ... ... ... ... ... ... ... ... ... [420]

#AF417713 ... ... ... ... ... ... ... ... ... ... ... ... ... ... ... ... ... ... ... ... [420]

#KR817728 ... ... ... ... ... ... ... ... ... ... ... ... ... ... ... ... ... ... ... ... [420]

#MW012492 CAC TGA TAC CCT TTA TTA ACA GGA TTA ACT ATA AAC CCT ACA TGA TTA AAA ATC CAA TTT [480]

#MZ269698 ... ... ... ... ... ... ... ... ... ... ... ... ... ... ... ... ... ... ... ... [480]

#MZ269699 ... ... ... ... ... ... ... ... ... ... ... ... ... ... ... ... ... ... ... ... [480]

#MZ269700 ... ... ... ... ... ... ... ... ... ... ... ... ... ... ... ... ... ... ... ... [480]

#MZ269701 ... ... ... ... ... ... ... ... ... ... ... ... ... ... ... ... ... ... ... ... [480]

#MZ269702 ... ... ... ... ... ... ... ... ... ... ... ... ... ... ... ... ... ... ... ... [480]

#MZ269703 ... ... ... ... ... ... ... ... ... ... ... ... ... ... ... ... ... ... ... ... [480]

#MZ269704 ... ... ... ... ... ... ... ... ... ... ... ... ... ... ... ... ... ... ... ... [480]

#MZ269705 ... ... ... ... ... ... ... ... ... ... ... ... ... ... ... ... ... ... ... ... [480]

#KT899888 ... ... ... ... ... ... ... ... ... ... ... ... ... ... ... ... ... ... ... ... [480]

#AY877426 ... ... ... ... ... ... ... ... ... ... ... ... ... ... ... ... ... ... ... ... [480]

#AY877428 ... ... ... ... ... ... ... ... ... ... ... ... ... ... ... ... ... ... ... ... [480]

#AY877429 ... ... ... ... ... ... ... ... ... ... ... ... ... ... ... ... ... ... ... ... [480]

#AY877427 ... ... ... ... ... ... ... ... ... ... ... ... ... ... ... ... ... ... ... ... [480]

#AF417713 ... ... ... ... ... ... ... ... ... ... ... ... ... ... ... ... ... ... ... ... [480]

#KR817728 ... ... ... ... ... ... ... ... ... ... ... ... ... ... ... ... ... ... ... ... [480]

#MW012492 TCT ATT ATA TTT ATT GGA GTA AAT TTA ACA TTT TTC CCG CAA CAT TTT CTA GGA TTA GCC [540]

#MZ269698 ... ... ... ... ... ... ... ... ... ... ... ... ... ... ... ... ... ... ... ... [540]

#MZ269699 ... ... ... ... ... ... ... ... ... ... ... ... ... ... ... ... ... ... ... ... [540]

#MZ269700 ... ... ... ... ... ... ... ... ... ... ... ... ... ... ... ... ... ... ... ... [540]

#MZ269701 ... ... ... ... ... ... ... ... ... ... ... ... ... ... ... ... ... ... ... ... [540]

#MZ269702 ... ... ... ... ... ... ... ... ... ... ... ... ... ... ... ... ... ... ... ... [540]

#MZ269703 ... ... ... ... ... ... ... ... ... ... ... ... ... ... ... ... ... ... ... ... [540]

#MZ269704 ... ... ... ... ... ... ... ... ... ... ... ... ... ... ... ... ... ... ... ... [540]

#MZ269705 ... ... ... ... ... ... ... ... ... ... ... ... ... ... ... ... ... ... ... ... [540]

#KT899888 ... ... ... ... ... ... ... ... ... ... ... ... ... ... ... ... ... ... ... ... [540]

#AY877426 ... ... ... ... ... ... ... ... ... ... ... ... ..A ... ... ... ... ... ... ... [540]

#AY877428 ... ... ... ... ... ... ... ... ... ... ... ... ..A ... ... ... ... ... ... ... [540]

#AY877429 ... ... ... ... ... ... ... ... ... ... ... ... ..A ... ... ... ... ... ... ... [540]

#AY877427 ... ... ... ... ... ... ... ... ... ... ... ... ..A ... ... ... ... ... ... ... [540]

#AF417713 ... ... ... ... ... ... ... ... ... ... ... ... ... ... ... ... ... ... ... ... [540]

#KR817728 ... ... ... ... ... ... ... ... ... ... ... ... ..A ... ... ... ... ... ... ... [540]

#MW012492 GGA ATA CCT CGA CGA TAT TCA GAT TTT CCA GAT AGT TAT CTA TCT TGA AAT ATT ATT TCT [600]

#MZ269698 ... ... ... ... ... ... ... ... ... ... ... ... ... ... ... ... ... ... ... ... [600]

#MZ269699 ... ... ... ... ... ... ... ... ... ... ... ... ... ... ... ... ... ... ... ... [600]

#MZ269700 ... ... ... ... ... ... ... ... ... ... ... ... ... ... ... ... ... ... ... ... [600]

#MZ269701 ... ... ... ... ... ... ... ... ... ... ... ... ... ... ... ... ... ... ... ... [600]

#MZ269702 ... ... ... ... ... ... ... ... ... ... ... ... ... ... ... ... ... ... ... ... [600]

#MZ269703 ... ... ... ... ... ... ... ... ... ... ... ... ... ... ... ... ... ... ... ... [600]

#MZ269704 ... ... ... ... ... ... ... ... ... ... ... ... ... ... ... ... ... ... ... ... [600]

#MZ269705 ... ... ... ... ... ... ... ... ... ... ... ... ... ... ... ... ... ... ... ... [600]

#KT899888 ... ... ... ... ... ... ... ... ... ... ... ... ... ... ... ... ... ... ... ... [600]

#AY877426 ... ... ... ... ... ..C ... ... ... ... ... ... ... ... ... ... ... ... ... ... [600]

#AY877428 ... ... ... ... ... ..C ... ... ... ... ... ... ... ... ... ... ... ... ... ... [600]

#AY877429 ... ... ... ... ... ..C ... ... ... ... ... ... ... ... ... ... ... ... ... ... [600]

#AY877427 ... ... ... ... ... ..C ... ... ... ... ... ... ... ... ... ... ... ... ... ... [600]

#AF417713 ... ... ... ... ... ... ... ... ... ... ... ... ... ... ... ... ... ... ... ... [600]

#KR817728 ... ... ... ... ... ..C ... ... ... ... ... ... ... ... ... ... ... ... ... ... [600]

#MW012492 TCA TTA GGA AGT ACA ATC TCA TTA TTT GCT ATT TTG TAT TTT TTA TTT ATT ATT TGA GAA [660]

#MZ269698 ... ... ... ... ... ... ... ... ... ... ... ... ... ... ... ... ... ... ... ... [660]

#MZ269699 ... ... ... ... ... ... ... ... ... ... ... ... ... ... ... ... ... ... ... ... [660]

#MZ269700 ... ... ... ... ... ... ... ... ... ... ... ... ... ... ... ... ... ... ... ... [660]

#MZ269701 ... ... ... ... ... ... ... ... ... ... ... ... ... ... ... ... ... ... ... ... [660]

#MZ269702 ... ... ... ... ... ... ... ... ... ... ... ... ... ... ... ... ... ... ... ... [660]

#MZ269703 ... ... ... ... ... ... ... ... ... ... ... ... ... ... ... ... ... ... ... ... [660]

#MZ269704 ... ... ... ... ... ... ... ... ... ... ... ... ... ... ... ... ... ... ... ... [660]

#MZ269705 ... ... ... ... ... ... ... ... ... ... ... ... ... ... ... ... ... ... ... ... [660]

#KT899888 ... ... ... ... ... ... ... ... ... ... ... ... ... ... ... ... ... ... ... ... [660]

#AY877426 ... ... ... ... ... ... ... ... ... ... ... ..A ... ... ... ... ... ... ... ... [660]

#AY877428 ... ... ... ... ... ... ... ... ... ... ... ..A ... ... ... ... ... ... ... ... [660]

#AY877429 ... ... ... ... ... ... ... ... ... ... ... ..A ... ... ... ... ... ... ... ... [660]

#AY877427 ... ... ... ... ... ... ... ... ... ... ... ..A ... ... ... ... ... ... ... ... [660]

#AF417713 ... ... ... ... ... ... ... ... ... ... ... ... ... ... ... ... ... ... ... ... [660]

#KR817728 ... ... ... ... ... ... ... ... ... ... ... ..A ... ... ... ... ... ... ... ... [660]

#MW012492 AGT ATA ATT ACA CAA CGT ACA CCT AGT TTT CCT ATA CAA TTG TCT TCA TCA ATT GAA TGA [720]

#MZ269698 ... ... ... ... ... ... ... ... ... ... ... ... ... ... ... ... ... ... ... ... [720]

#MZ269699 ... ... ... ... ... ... ... ... ... ... ... ... ... ... ... ... ... ... ... ... [720]

#MZ269700 ... ... ... ... ... ... ... ... ... ... ... ... ... ... ... ... ... ... ... ... [720]

#MZ269701 ... ... ... ... ... ... ... ... ... ... ... ... ... ... ... ... ... ... ... ... [720]

#MZ269702 ... ... ... ... ... ... ... ... ... ... ... ... ... ... ... ... ... ... ... ... [720]

#MZ269703 ... ... ... ... ... ... ... ... ... ... ... ... ... ... ... ... ... ... ... ... [720]

#MZ269704 ... ... ... ... ... ... ... ... ... ... ... ... ... ... ... ... ... ... ... ... [720]

#MZ269705 ... ... ... ... ... ... ... ... ... ... ... ... ... ... ... ... ... ... ... ... [720]

#KT899888 ... ... ... ... ... ... ... ... ... ... ... ... ... ... ... ... ... ... ... ... [720]

#AY877426 ... ... ... ... ... ... ... ... ... ... ... ... ... ..A ... ... ... ... ... ... [720]

#AY877428 ... ... ... ... ... ... ... ... ... ... ... ... ... ..A ... ... ... ... ... ... [720]

#AY877429 ... ... ... ... ... ... ... ... ... ... ... ... ... ..A ... ... ... ... ... ... [720]

#AY877427 ... ... ... ... ... ... ... ... ... ... ... ... ... ..A ... ... ... ... ... ... [720]

#AF417713 ... ... ... ... ... ... ... ... ... ... ... ... ... ... ... ... ... ... ... ... [720]

#KR817728 ... ... ... ... ... ... ... ... ... ... ... ... ... ..A ... ... ... ... ... ... [720]

#MW012492 TAT CAT ACC CTT CCC CCA GCT GAG CAT ACA TAT GCA GA [758]

#MZ269698 ... ... ... ... ... ... ... ... ... ... ... ... .. [758]

#MZ269699 ... ... ... ... ... ... ... ... ... ... ... ... .. [758]

#MZ269700 ... ... ... ... ... ... ... ... ... ... ... ... .. [758]

#MZ269701 ... ... ... ... ... ... ... ... ... ... ... ... .. [758]

#MZ269702 ... ... ... ... ... ... ... ... ... ... ... ... .. [758]

#MZ269703 ... ... ... ... ... ... ... ... ... ... ... ... .. [758]

#MZ269704 ... ... ... ... ... ... ... ... ... ... ... ... .. [758]

#MZ269705 ... ... ... ... ... ... ... ... ... ... ... ... .. [758]

#KT899888 ... ... ... ... ... ... ... ... ... ... ... ... .. [758]

#AY877426 ... ... ..T ... ... ... ... ... ... ... ... ... .. [758]

#AY877428 ... ... ..T ... ... ... ... ... ... ... ... ... .. [758]

#AY877429 ... ... ..T ... ... ... ... ... ... ... ... ... .. [758]

#AY877427 ... ... ..T ... ... ... ... ... ... ... ... ... .. [758]

#AF417713 ... ... ... ... ... ... ... ... ... ... ... ... .. [758]

#KR817728 ... ... ..T ... ... ... ... ... ... ... ... ... .. [758]
