# Supplementary material for: Identification of a biological form in the Anopheles stephensi laboratory colony using the odorant-binding protein 1 intron I sequence
Source: PLoS One. 2022 Feb 22;17(2):e0263836. doi: 10.1371/journal.pone.0263836 (PMC8863247; doi:10.1371/journal.pone.0263836)
Supplement: S2 Fig — Bootstrap values >70 shown at nodes. (DOCX) [file pone.0263836.s002.docx]

#MZ420723 TTC TTC ACC TTT AAT AGA ACA ATT AAA TTT TTT TCA TGA TCA TAC ACT TCT TAT TTT AAC [ 60]

#MZ420724 ... ... ... ... ... ... ... ... ... ... ... ... ... ... ... ... ... ... ... ... [ 60]

#MZ420725 ... ... ... ... ... ... ... ... ... ... ... ... ... ... ... ... ... ... ... ... [ 60]

#MZ420726 ... ... ... ... ... ... ... ... ... ... ... ... ... ... ... ... ... ... ... ... [ 60]

#MZ420727 ... ... ... ... ... ... ... ... ... ... ... ... ... ... ... ... ... ... ... ... [ 60]

#MZ420728 ... ... ... ... ... ... ... ... ... ... ... ... ... ... ... ... ... ... ... ... [ 60]

#MZ420729 ... ... ... ... ... ... ... ... ... ... ... ... ... ... ... ... ... ... ... ... [ 60]

#MZ420730 ... ... ... ... ... ... ... ... ... ... ... ... ... ... ... ... ... ... ... ... [ 60]

#MW431057 ... ... ... ... ... ... ... ... ... ... ... ... ... ... ... ... ... ... ... ... [ 60]

#KT899888 ... ... ... ... ... ... ... ... ... ... ... ... ... ... ... ... ... ... ... ... [ 60]

#AF425844 ... ... ... ... ... ... ... ... ... ... ... ... ... ... ... ... ... ... ... ... [ 60]

#DQ022847 ... ... ... ... ... ... ... ... ... ... ... ... ... ... ... ... ... ... ... ... [ 60]

#DQ022846 ... ... ... ... ... ... ... ... ... ... ... ... ... ... ... ... ... ... ... ... [ 60]

#DQ022845 ... ... ... ... ... ... ... ... ... ... ... ... ... ... ... ... ... ... ... ... [ 60]

#DQ026675 ... ... ... ... ... ... ... ... ... ... ... ... ... ... ... ... ... ... ... ... [ 60]

#AY883832 ... ... ... ... ... ... ... ... ... ... ... ... ... ... ... ... ... ... ... ... [ 60]

#AY883830 ... ... ... ... ... ... ... ... ... ... ... ... ... ... ... ... ... ... ... ... [ 60]

#AY883837 ... ... ... ... ... ... ... ... ... ... ... ... ... ... ... ... ... ... ... ... [ 60]

#AY883836 ... ... ... ... ... ... ... ... ... ... ... ... ... ... ... ... ... ... ... ... [ 60]

#AY883835 ... ... ... ... ... ... ... ... ... ... ... ... ... ... ... ... ... ... ... ... [ 60]

#AY883834 ... ... ... ... ... ... ... ... ... ... ... ... ... ... ... ... ... ... ... ... [ 60]

#AY883833 ... ... ... ... ... ... ... ... ... ... ... ... ... ... ... ... ... ... ... ... [ 60]

#AY883831 ... ... ... ... ... ... ... ... ... ... ... ... ... ... ... ... ... ... ... ... [ 60]

#KY863454 ... ... ... ... ... ... ... ... ... ... ... ... ... ... ... ... ... ... ... ... [ 60]

#EF208912 ... ... .G. ... G.. ... ... ... ... ... ... ... ... ... ... ... ... ... ... ... [ 60]

#FJ526438 ... ... ... ... ... ... ... ... ... ... ... ... ... ... ... ... ... ... ... ... [ 60]

#FJ526437 ... ... ... ... ... ... ... ... ... ... ... ... ... ... ... ... ... ... ... ... [ 60]

#AF417749 ... ... ... ... ... ... ... ... ... ... ... ... ... ... ... ... ... ... ... ... [ 60]

#JX139608 ... ... ... ... ... ... ... ... ... ... ... ... ... ... ... ... ... ... ... ... [ 60]

#MZ420723 TAT AAT TAC AAT TTT AGT AGG ATA TAT TAT AGG AAT ATT AAT ATT TAA TAA ATT TAC TAA [120]

#MZ420724 ... ... ... ... ... ... ... ... ... ... ... ... ... ... ... ... ... ... ... ... [120]

#MZ420725 ... ... ... ... ... ... ... ... ... ... ... ... ... ... ... ... ... ... ... ... [120]

#MZ420726 ... ... ... ... ... ... ... ... ... ... ... ... ... ... ... ... ... ... ... ... [120]

#MZ420727 ... ... ... ... ... ... ... ... ... ... ... ... ... ... ... ... ... ... ... ... [120]

#MZ420728 ... ... ... ... ... ... ... ... ... ... ... ... ... ... ... ... ... ... ... ... [120]

#MZ420729 ... ... ... ... ... ... ... ... ... ... ... ... ... ... ... ... ... ... ... ... [120]

#MZ420730 ... ... ... ... ... ... ... ... ... ... ... ... ... ... ... ... ... ... ... ... [120]

#MW431057 ... ... ... ... ... ... ... ... ... ... ... ... ... ... ... ... ... ... ... ... [120]

#KT899888 ... ... ... ... ... ... ... ... ... ... ... ... ... ... ... ... ... ... ... ... [120]

#AF425844 ... ... ... ... ... ... ... ... ... ... ... ... ... ... ... ... ... ... ... ... [120]

#DQ022847 ... ... ... ... ... ... ... ... ... ... ... ... ... ... ... ... ... ... ... ... [120]

#DQ022846 ... ... ... ... ... ... ... ... ... ... ... ... ... ... ... ... ... ... ... ... [120]

#DQ022845 ... ... ... ... ... ... ... ... ... ... ... ... ... ... ... ... ... ... ... ... [120]

#DQ026675 ... ... ... ... ... ... ... ... ... ... ... ... ... ... ... ... ... ... ... ... [120]

#AY883832 ... ... ... ... ... ... ... ... ... ... ... ... ... ... ... ... ... ... ... ... [120]

#AY883830 ... ... ... ... ... ... ... ... ... ... ... ... ... ... ... ... ... ... ... ... [120]

#AY883837 ... ... ... ... ... ... ... ... ... ... ... ... ... ... ... ... ... ... ... ... [120]

#AY883836 ... ... ... ... ... ... ... ... ... ... ... ... ... ... ... ... ... ... ... ... [120]

#AY883835 ... ... ... ... ... ... ... ... ... ... ... ... ... ... ... ... ... ... ... ... [120]

#AY883834 ... ... ... ... ... ... ... ... ... ... ... ... ... ... ... ... ... ... ... ... [120]

#AY883833 ... ... ... ... ... ... ... ... ... ... ... ... ... ... ... ... ... ... ... ... [120]

#AY883831 ... ... ... ... ... ... ... ... ... ... ... ... ... ... ... ... ... ... ... ... [120]

#KY863454 ... ... ... ... ... ... ... ... ... ... ... ... ... ... ... ... ... ... ... ... [120]

#EF208912 ... ... ... ... ... ... ... ... ... ... ... ... ... ... ... ... ... ... ... ... [120]

#FJ526438 ... ... ... ... ... ... ... ... ... ... ... ... ... ... ... ... ... ... ... ... [120]

#FJ526437 ... ... ... ... ... ... ... ... ... ... ... ... ... ... ... ... ... ... ... ... [120]

#AF417749 ... ... ... ... ... ... ... ... ... ... ... ... ... ... ... ... ... ... ... ... [120]

#JX139608 ... ... ... ... ... ... ... ... ... ... ... ... ... ... ... ... ... ... ... ... [120]

#MZ420723 TCG ATT TTT ACT TCA TGG ACA AAC AAT TGA AAT TAT TTG AAC AGT TCT ACC AGC AAT TAT [180]

#MZ420724 ... ... ... ... ... ... ... ... ... ... ... ... ... ... ... ... ... ... ... ... [180]

#MZ420725 ... ... ... ... ... ... ... ... ... ... ... ... ... ... ... ... ... ... ... ... [180]

#MZ420726 ... ... ... ... ... ... ... ... ... ... ... ... ... ... ... ... ... ... ... ... [180]

#MZ420727 ... ... ... ... ... ... ... ... ... ... ... ... ... ... ... ... ... ... ... ... [180]

#MZ420728 ... ... ... ... ... ... ... ... ... ... ... ... ... ... ... ... ... ... ... ... [180]

#MZ420729 ... ... ... ... ... ... ... ... ... ... ... ... ... ... ... ... ... ... ... ... [180]

#MZ420730 ... ... ... ... ... ... ... ... ... ... ... ... ... ... ... ... ... ... ... ... [180]

#MW431057 ... ... ... ... ... ... ... ... ... ... ... ... ... ... ... ... ... ... ... ... [180]

#KT899888 ... ... ... ... ... ... ... ... ... ... ... ... ... ... ... ... ... ... ... ... [180]

#AF425844 ... ... ... ... ... ... ... ... ... ... ... ... ... ... .A. ... ... ... ... ... [180]

#DQ022847 ... ... ... ... ... ... ... ... ... ... ... ... ... ... ... ... ... ... ... ... [180]

#DQ022846 ... ... ... ... ... ... ... ... ... ... ... ... ... ... ... ... ... ... ... ... [180]

#DQ022845 ... ... ... ... ... ... ... ... ... ... ... ... ... ... ... .T. ... ... ... ... [180]

#DQ026675 ... ... ... ... ... ... ... ... ... ... ... ... ... ... .A. ... ... ... ... ... [180]

#AY883832 ... ... ... ... ... ... ... ... ... ... ... ... ... ... ... .T. ... ... ... ... [180]

#AY883830 ... ... ... ... ... ... ... ... ... ... ... ... ... ... ... .T. ... ... ... ... [180]

#AY883837 ... ... ... ... ... ... ... ... ... ... ... ... ... ... ... .T. ... ... ... ... [180]

#AY883836 ... ... ... ... ... ... ... ... ... ... ... ... ... ... ... .T. ... ... ... ... [180]

#AY883835 ... ... ... ... ... ... ... ... ... ... ... ... ... ... ... .T. ... ... ... ... [180]

#AY883834 ... ... ... ... ... ... ... ... ... ... ... ... ... ... ... .T. ... ... ... ... [180]

#AY883833 ... ... ... ... ... ... ... ... ... ... ... ... ... ... ... .T. ... ... ... ... [180]

#AY883831 ... ... ... ... ... ... ... ... ... ... ... ... ... ... ... .T. ... ... ... ... [180]

#KY863454 ... ... ... ... ... ... ... ... ... ... ... ... ... ... ... .T. ... ... ... ... [180]

#EF208912 ... ... ... ... ... ... ... ... ... ... ... ... ... ... ... ... ... ... ... ... [180]

#FJ526438 ... ... ... ... ... ... ... ... ... ... ... ... ... ... ... .T. ... ... ... ... [180]

#FJ526437 ... ... ... ... ... ... ... ... ... ... ... ... ... ... ... ... ... ... ... ... [180]

#AF417749 ... ... ... ... ... ... ... ... ... ... ... ... ... ... .A. ... ... ... ... ... [180]

#JX139608 ... ... ... ... ... ... ... ... ... ... ... ... ... ... ... ... ... ... ... ... [180]

#MZ420723 TTT AAT ATT TAT TGC CTT TCC ATC TTT ACG ATT ATT ATA TTT AAT AGA TGA AAT TAA TAC [240]

#MZ420724 ... ... ... ... ... ... ... ... ... ... ... ... ... ... ... ... ... ... ... ... [240]

#MZ420725 ... ... ... ... ... ... ... ... ... ... ... ... ... ... ... ... ... ... ... ... [240]

#MZ420726 ... ... ... ... ... ... ... ... ... ... ... ... ... ... ... ... ... ... ... ... [240]

#MZ420727 ... ... ... ... ... ... ... ... ... ... ... ... ... ... ... ... ... ... ... ... [240]

#MZ420728 ... ... ... ... ... ... ... ... ... ... ... ... ... ... ... ... ... ... ... ... [240]

#MZ420729 ... ... ... ... ... ... ... ... ... ... ... ... ... ... ... ... ... ... ... ... [240]

#MZ420730 ... ... ... ... ... ... ... ... ... ... ... ... ... ... ... ... ... ... ... ... [240]

#MW431057 ... ... ... ... ..T ... ... ... ... ... ... ... ... ... ... ... ... ... ... ... [240]

#KT899888 ... ... ... ... ... ... ... ... ... ... ... ... ... ... ... ... ... ... ... ... [240]

#AF425844 ... ... ... ... ... ... ... ... ... ... ... ... ... ... ... ... ... ... ... ... [240]

#DQ022847 ... ... ... ... ... ... ... ... ... ... ... ... ... ... ... ... ... ... ... ... [240]

#DQ022846 ... ... ... ... ... ... ... ... ... ... ... ... ... ... ... ... ... ... ... ... [240]

#DQ022845 ... ... ... ... ... ... ... ... ... ... ... ... ... ... ... ... ... ... ... ... [240]

#DQ026675 ... ... ... ... ... ... ... ... ... ... ... ... ... ... ... ... ... ... ... ... [240]

#AY883832 ... ... ... ... ... ... ... ... ... ... ... ... ... ... ... ... ... ... ... ... [240]

#AY883830 ... ... ... ... ... ... ... ... ... ... ... ... ... ... ... ... ... ... ... ... [240]

#AY883837 ... ... ... ... ... ... ... ... ... ... ... ... ... ... ... ... ... ... ... ... [240]

#AY883836 ... ... ... ... ... ... ... ... ... ... ... ... ... ... ... ... ... ... ... ... [240]

#AY883835 ... ... ... ... ... ... ... ... ... ... ... ... ... ... ... ... ... ... ... ... [240]

#AY883834 ... ... ... ... ... ... ... ... ... ... ... ... ... ... ... ... ... ... ... ... [240]

#AY883833 ... ... ... ... ... ... ... ... ... ... ... ... ... ... ... ... ... ... ... ... [240]

#AY883831 ... ... ... ... ... ... ... ... ... ... ... ... ... ... ... ... ... ... ... ... [240]

#KY863454 ... ... ... ... ... ... ... ... ... ... ... ... ... ... ... ... ... ... ... ... [240]

#EF208912 ... ... ... ... ... ... ... ... ... ... ... ... ... ... ... ... ... ... ... ... [240]

#FJ526438 ... ... ... ... ... ... ... ... ... ... ... ... ... ... ... ... ... ... ... ... [240]

#FJ526437 ... ... ... ... ... ... ... ... ... ... ... ... ... ... ... ... ... ... ... ... [240]

#AF417749 ... ... ... ... ... ... ... ... ... ... ... ... ... ... ... ... ... ... ... ... [240]

#JX139608 ... ... ... ... ... ... ... ... ... ... ... ... ... ... ... ... ... ... ... ... [240]

#MZ420723 TCC ATC TAT TAC CTT AAA ATC AAT TGG ACA TCA GTG ATA CTG AAG ATA TGA ATA TTC TGA [300]

#MZ420724 ... ... ... ... ... ... ... ... ... ... ... ... ... ... ... ... ... ... ... ... [300]

#MZ420725 ... ... ... ... ... ... ... ... ... ... ... ... ... ... ... ... ... ... ... ... [300]

#MZ420726 ... ... ... ... ... ... ... ... ... ... ... ... ... ... ... ... ... ... ... ... [300]

#MZ420727 ... ... ... ... ... ... ... ... ... ... ... ... ... ... ... ... ... ... ... ... [300]

#MZ420728 ... ... ... ... ... ... ... ... ... ... ... ... ... ... ... ... ... ... ... ... [300]

#MZ420729 ... ... ... ... ... ... ... ... ... ... ... ... ... ... ... ... ... ... ... ... [300]

#MZ420730 ... ... ... ... ... ... ... ... ... ... ... ... ... ... ... ... ... ... ... ... [300]

#MW431057 ... ... ... ... ... ... ... ... ... ... ... ... ... ... ... ... ... ... ... ... [300]

#KT899888 ... ... ... ... ... ... ... ... ... ... ... ... ... ... ... ... ... ... ... ... [300]

#AF425844 ... ... ... ... ... ... ... ... ... ... ... ... ... ... ... ... ... ... ... ... [300]

#DQ022847 ... ... ... ... ... ... ... ... ... ... ... ... ... ... ... ... ... ... ... ... [300]

#DQ022846 ... ... ... ... ... ... ... ... ... ... ... ... ... ... ... ... ... ... ... ... [300]

#DQ022845 ... ... ... ... T.. ... ... ... ... ... ... ... ... ... ... ... ... ... ... ... [300]

#DQ026675 ... ... ... ... ... ... ... ... ... ... ... ... ... ... ... ... ... ... ... ... [300]

#AY883832 ... ... ... ... T.. ... ... ... ... ... ... ... ... ... ... ... ... ... ... ... [300]

#AY883830 ... ... ... ... T.. ... ... ... ... ... ... ... ... ... ... ... ... ... ... ... [300]

#AY883837 ... ... ... ... T.. ... ... ... ... ... ... ... ... ... ... ... ... ... ... ... [300]

#AY883836 ... ... ... ... T.. ... ... ... ... ... ... ... ... ... ... ... ... ... ... ... [300]

#AY883835 ... ... ... ... T.. ... ... ... ... ... ... ... ... ... ... ... ... ... ... ... [300]

#AY883834 ... ... ... ... T.. ... ... ... ... ... ... ... ... ... ... ... ... ... ... ... [300]

#AY883833 ... ... ... ... T.. ... ... ... ... ... ... ... ... ... ... ... ... ... ... ... [300]

#AY883831 ... ... ... ... T.. ... ... ... ... ... ... ... ... ... ... ... ... ... ... ... [300]

#KY863454 ... ... ... ... T.. ... ... ... ... ... ... ... ... ... ... ... ... ... ... ... [300]

#EF208912 ... ... ... ... ... ... ... ... ... ... ... ... ... ... ... ... ... ... ... ... [300]

#FJ526438 ... ... ... ... T.. ... ... ... ... ... ... ... ... ... ... ... ... ... ... ... [300]

#FJ526437 ... ... ... ... ... ... ... ... ... ... ... ... ... ... ... ... ... ... ... ... [300]

#AF417749 ... ... ... ... ... ... ... ... ... ... ... ... ... ... ... ... ... ... ... ... [300]

#JX139608 ... ... ... ... ... ... ... ... ... ... ... ... ... ... ... ... ... ... ... ... [300]

#MZ420723 TTT TTT AAA TTT AGA ATT TGA TTC ATA TAT AAT TCC TAC TAA TGA ATT AGA AAC AAA CGG [360]

#MZ420724 ... ... ... ... ... ... ... ... ... ... ... ... ... ... ... ... ... ... ... ... [360]

#MZ420725 ... ... ... ... ... ... ... ... ... ... ... ... ... ... ... ... ... ... ... ... [360]

#MZ420726 ... ... ... ... ... ... ... ... ... ... ... ... ... ... ... ... ... ... ... ... [360]

#MZ420727 ... ... ... ... ... ... ... ... ... ... ... ... ... ... ... ... ... ... ... ... [360]

#MZ420728 ... ... ... ... ... ... ... ... ... ... ... ... ... ... ... ... ... ... ... ... [360]

#MZ420729 ... ... ... ... ... ... ... ... ... ... ... ... ... ... ... ... ... ... ... ... [360]

#MZ420730 ... ... ... ... ... ... ... ... ... ... ... ... ... ... ... ... ... ... ... ... [360]

#MW431057 ... ... ... ... ... ... ... ... ... ... ... ... ... ... ... ... ... ... ... ... [360]

#KT899888 ... ... ... ... ... ... ... ... ... ... ... ... ... ... ... ... ... ... ... ... [360]

#AF425844 ... ... ... ... ... ... ... ... ... ... ... ... ... ... ... ... ... ... ... ... [360]

#DQ022847 ... ... ... ... ... ... ... ... ... ... ... ... ... ... ... ... ... ... ... ... [360]

#DQ022846 ... ... ... ... ... ... ... ... ... ... ... ... ... ... ... ... ... ... ... ... [360]

#DQ022845 ... ... ... ... ... ... ... ... G.. ... ... ... ... ... ... ... ... ... ... ... [360]

#DQ026675 ... ... ... ... ... ... ... ... ... ... ... ... ... ... ... ... ... ... ... ... [360]

#AY883832 ... ... ... ... ... ... ... ... G.. ... ... ... ... ... ... ... ... ... ... ... [360]

#AY883830 ... ... ... ... ... ... ... ... G.. ... ... ... ... ... ... ... ... ... ... ... [360]

#AY883837 ... ... ... ... ... ... ... ... G.. ... ... ... ... ... ... ... ... ... ... ... [360]

#AY883836 ... ... ... ... ... ... ... ... G.. ... ... ... ... ... ... ... ... ... ... ... [360]

#AY883835 ... ... ... ... ... ... ... ... G.. ... ... ... ... ... ... ... ... ... ... ... [360]

#AY883834 ... ... ... ... ... ... ... ... G.. ... ... ... ... ... ... ... ... ... ... ... [360]

#AY883833 ... ... ... ... ... ... ... ... G.. ... ... ... ... ... ... ... ... ... ... ... [360]

#AY883831 ... ... ... ... ... ... ... ... G.. ... ... ... ... ... ... ... ... ... ... ... [360]

#KY863454 ... ... ... ... ... ... ... ... G.. ... ... ... ... ... ... ... ... ... ... ... [360]

#EF208912 ... ... ... ... ... ... ... ... ... ... ... ... ... ... ... ... ... ... ... ... [360]

#FJ526438 ... ... ... ... ... ... ... ... G.. ... ... ... ... ... ... ... ... ... ... ... [360]

#FJ526437 ... ... ... ... ... ... ... ... ... ... ... ... ... ... ... ... ... ... ... ... [360]

#AF417749 ... ... ... ... ... ... ... ... ... ... ... ... ... ... ... ... ... ... ... ... [360]

#JX139608 ... ... ... ... ... ... ... ... ... ... ... ... ... ... ... ... ... ... ... ... [360]

#MZ420723 ATT TCG ATT ATT AGA TGT TGA TAA TCG AGT TGT TTT ACC AAT AAA CAA TCA AAT TCG AAT [420]

#MZ420724 ... ... ... ... ... ... ... ... ... ... ... ... ... ... ... ... ... ... ... ... [420]

#MZ420725 ... ... ... ... ... ... ... ... ... ... ... ... ... ... ... ... ... ... ... ... [420]

#MZ420726 ... ... ... ... ... ... ... ... ... ... ... ... ... ... ... ... ... ... ... ... [420]

#MZ420727 ... ... ... ... ... ... ... ... ... ... ... ... ... ... ... ... ... ... ... ... [420]

#MZ420728 ... ... ... ... ... ... ... ... ... ... ... ... ... ... ... ... ... ... ... ... [420]

#MZ420729 ... ... ... ... ... ... ... ... ... ... ... ... ... ... ... ... ... ... ... ... [420]

#MZ420730 ... ... ... ... ... ... ... ... ... ... ... ... ... ... ... ... ... ... ... ... [420]

#MW431057 ... ... ... ... ... ... ... ... ... ... ... ... ... ... ... ... ... ... ... ... [420]

#KT899888 ... ... ... ... ... ... ... ... ... ... ... ... ... ... ... ... ... ... ... ... [420]

#AF425844 ... ... ... ... ... ... ... ... ... ... ... ... ... ... ... ... ... ... ... ... [420]

#DQ022847 ... ... ... ... ... ... ... ... ... ... ... ... ... ... ... ... ... ... ... ... [420]

#DQ022846 ... ... ... ... ... ... ... ... ... ... ... ... ... ... ... ... ... ... ... ... [420]

#DQ022845 G.. ... ... G.. ... ... ... ... ... ... ... ... ... ... ... ... ... ... ... ... [420]

#DQ026675 ... ... ... ... ... ... ... ... ... ... ... ... ... ... ... ... ... ... ... ... [420]

#AY883832 G.. ... ... G.. ... ... ... ... ... ... ... ... ... ... ... ... ... ... ... ... [420]

#AY883830 G.. ... ... G.. ... ... ... ... ... ... ... ... ... ... ... ... ... ... ... ... [420]

#AY883837 G.. ... ... G.. ... ... ... ... ... ... ... ... ... ... ... ... ... ... ... ... [420]

#AY883836 G.. ... ... G.. ... ... ... ... ... ... ... ... ... ... ... ... ... ... ... ... [420]

#AY883835 G.. ... ... G.. ... ... ... ... ... ... ... ... ... ... ... G.. ... ... ... ... [420]

#AY883834 G.. ... ... G.. ... ... ... ... ... ... ... ... ... ... ... ... ... ... ... ... [420]

#AY883833 G.. ... ... G.. ... ... ... ... ... ... ... ... ... ... ... ... ... ... ... ... [420]

#AY883831 G.. ... ... G.. ... ... ... ... ... ... ... ... ... ... ... ... ... ... ... ... [420]

#KY863454 G.. ... ... G.. ... ... ... ... ... ... ... ... ... ... ... ... ... ... ... ... [420]

#EF208912 ... ... ... ... ... ... ... ... ... ... ... ... ... ... ... ... ... ... ... ... [420]

#FJ526438 G.. ... ... G.. ... ... ... ... ... ... ... ... ... ... ... ... ... ... ... ... [420]

#FJ526437 ... ... ... ... ... ... ... ... ... ... ... ... ... ... ... ... ... ... ... ... [420]

#AF417749 ... ... ... ... ... ... ... ... ... ... ... ... ... ... ... ... ... ... ... ... [420]

#JX139608 ... ... ... ... ... ... ... ... ... ... ... ... ... ... ... ... ... ... ... ... [420]

#MZ420723 TTT AGT AAC AGC TAC TGA TGT ATT ACA CTC ATG AAC TGT TCC CTC TTT AGG AGT AAA GGT [480]

#MZ420724 ... ... ... ... ... ... ... ... ... ... ... ... ... ... ... ... ... ... ... ... [480]

#MZ420725 ... ... ... ... ... ... ... ... ... ... ... ... ... ... ... ... ... ... ... ... [480]

#MZ420726 ... ... ... ... ... ... ... ... ... ... ... ... ... ... ... ... ... ... ... ... [480]

#MZ420727 ... ... ... ... ... ... ... ... ... ... ... ... ... ... ... ... ... ... ... ... [480]

#MZ420728 ... ... ... ... ... ... ... ... ... ... ... ... ... ... ... ... ... ... ... ... [480]

#MZ420729 ... ... ... ... ... ... ... ... ... ... ... ... ... ... ... ... ... ... ... ... [480]

#MZ420730 ... ... ... ... ... ... ... ... ... ... ... ... ... ... ... ... ... ... ... ... [480]

#MW431057 ... ... ... ... ... ... ... ... ... ... G.. ... ... ... ... ... ... ... ... ... [480]

#KT899888 ... ... ... ... ... ... ... ... ... ... ... ... ... ... ... ... ... ... ... ... [480]

#AF425844 ... ... ... ... ... ... ... ... ... ... ... ... ... ... ... ... ... ... ... ... [480]

#DQ022847 ... ... ... ... ... ... ... ... ... ... ... ... ... ... ... ... ... ... ... ... [480]

#DQ022846 ... ... ... ... ... ... ... ... ... ... ... ... ... ... ... ... ... ... ... ... [480]

#DQ022845 ... ... ... ... ... ... ... ... ... ... ... ... ... ... ... ... ... ... ... ... [480]

#DQ026675 ... ... ... ... ... ... ... ... ... ... ... ... ... ... ... ... ... ... ... ... [480]

#AY883832 ... ... ... ... ... ... ... ... ... ... ... ... ... ... ... ... ... ... ... ... [480]

#AY883830 ... ... ... ... ... ... ... ... ... ... ... ... ... ... ... ... ... ... ... ... [480]

#AY883837 ... ... ... ... ... ... ... ... ... ... ... ... ... ... ... ... ... ... ... ... [480]

#AY883836 ... ... ... ... ... ... ... ... ... ... ... ... ... ... ... ... ... ... ... ... [480]

#AY883835 ... ... ... ... ... ... ... ... ... ... ... ... ... ... ... ... ... ... ... ... [480]

#AY883834 ... ... ... ... ... ... ... ... ... ... ... ... ... ... ... ... ... ... ... ... [480]

#AY883833 ... ... ... ... ... ... ... ... ... ... ... ... ... ... ... ... ... ... ... ... [480]

#AY883831 ... ... ... ... ... ... ... ... ... ... ... ... ... ... ... ... ... ... ... ... [480]

#KY863454 ... ... ... ... ... ... ... ... ... ... ... ... ... ... ... ... ... ... ... ... [480]

#EF208912 ... ... ... ... ... ... ... ... ... ... ... ... ... ... ... ... ... ... ... ... [480]

#FJ526438 ... ... ... ... ... ... ... ... ... ... ... ... ... ... ... ... ... ... ... ... [480]

#FJ526437 ... ... ... ... ... ... ... ... ... ... ... ... ... ... ... ... ... ... ... ... [480]

#AF417749 ... ... ... ... ... ... ... ... ... ... ... ... ... ... ... ... ... ... ... ... [480]

#JX139608 ... ... ... ... ... ... ... ... ... ... ... ... ... ... ... ... ... ... ... ... [480]

#MZ420723 AGA TGC TAC TCC TGG ACG ATT AAA TCA AAT TAA TTT CTT AAT TAA TCG ACC AGG ATT ATT [540]

#MZ420724 ... ... ... ... ... ... ... ... ... ... ... ... ... ... ... ... ... ... ... ... [540]

#MZ420725 ... ... ... ... ... ... ... ... ... ... ... ... ... ... ... ... ... ... ... ... [540]

#MZ420726 ... ... ... ... ... ... ... ... ... ... ... ... ... ... ... ... ... ... ... ... [540]

#MZ420727 ... ... ... ... ... ... ... ... ... ... ... ... ... ... ... ... ... ... ... ... [540]

#MZ420728 ... ... ... ... ... ... ... ... ... ... ... ... ... ... ... ... ... ... ... ... [540]

#MZ420729 ... ... ... ... ... ... ... ... ... ... ... ... ... ... A.. ... ... ... ... ... [540]

#MZ420730 ... ... ... ... ... ... ... ... ... ... ... ... ... ... ... ... ... ... ... ... [540]

#MW431057 ... ... ... ... ... ... ... ... ... ... ... ... ... ... ... ... ... ... ... ... [540]

#KT899888 ... ... ... ... ... ... ... ... ... ... ... ... ... ... ... ... ... ... ... ... [540]

#AF425844 ... ... ... ... ... ... ... ... ... ... ... ... ... ... ... ... ... ... ... ... [540]

#DQ022847 ... ... ... ... ... ... ... ... ... ... ... ... ... ... ... ... ... ... ... ... [540]

#DQ022846 ... ... ... ... ... ... ... ... ... ... ... ... ... ... ... ... ... ... ... ... [540]

#DQ022845 ... ... ... ... ... ... ... ... ... ... ... ... ... ... ... ... ... ... ... ... [540]

#DQ026675 ... ... ... ... ... ... ... ... ... ... ... ... ... ... ... ... ... ... ... ... [540]

#AY883832 ... ... ... ... ... ... ... ... ... ... ... ... ... ... ... ... ... ... ... ... [540]

#AY883830 ... ... ... ... ... ... ... ... ... ... ... ... ... ... ... ... ... ... ... ... [540]

#AY883837 ... ... ... ... ... ... ... ... ... ... ... ... ... ... ... ... ... ... ... ... [540]

#AY883836 ... ... ... ... ... ... ... ... ... ... ... ... ... ... ... ... ... ... ... ... [540]

#AY883835 ... ... ... ... ... ... ... ... ... ... ... ... ... ... ... ... ... ... ... ... [540]

#AY883834 ... ... ... ... ... ... ... ... ... ... ... ... ... ... ... ... ... ... ... ... [540]

#AY883833 ... ... ... ... ... ... ... ... ... ... ... ... ... ... ... ... ... ... ... ... [540]

#AY883831 ... ... ... ... ... ... ... ... ... ... ... ... ... ... ... ... ... ... ... ... [540]

#KY863454 ... ... ... ... ... ... ... ... ... ... ... ... ... ... ... ... ... ... ... ... [540]

#EF208912 ... ... ... ... ... ... ... ... ... ... ... ... ... ... ... ... ... ... ... ... [540]

#FJ526438 ... ... ... ... ... ... ... ... ... ... ... ... ... ... ... ... ... ... ... ... [540]

#FJ526437 ... ... ... ... ... ... ... ... ... ... ... ... ... ... ... ... ... ... ... ... [540]

#AF417749 ... ... ... ... ... ... ... ... ... ... ... ... ... ... ... ... ... ... ... ... [540]

#JX139608 ... ... ... ... ... ... ... ... ... ... ... ... ... ... ... ... ... ... ... ... [540]

#MZ420723 TTT TGG TCA ATG TTC AGA AA [560]

#MZ420724 ... ... ... ... ... ... .. [560]

#MZ420725 ... ... ... ... ... ... .. [560]

#MZ420726 ... ... ... ... ... ... .. [560]

#MZ420727 ... ... ... ... ... ... .. [560]

#MZ420728 ... ... ... ... ... ... .. [560]

#MZ420729 ... ... ... ... ... ... .. [560]

#MZ420730 ... ... ... ... ... ... .. [560]

#MW431057 ... ... ... ... ... ... .. [560]

#KT899888 ... ... A.. ... ... ... .. [560]

#AF425844 ... ... A.. ... ... ... .. [560]

#DQ022847 ... ... A.. ... ... ... .. [560]

#DQ022846 ... ... A.. ... ... ... .. [560]

#DQ022845 ... ... A.. ... ... ... .. [560]

#DQ026675 ... ... A.. ... ... ... .. [560]

#AY883832 ... ... A.. ... ... ... .. [560]

#AY883830 ... ... A.. ... ... ... .. [560]

#AY883837 ... ... A.. ... ... ... .. [560]

#AY883836 ... ... A.. ... ... ... .. [560]

#AY883835 ... ... A.. ... ... ... .. [560]

#AY883834 ... ... A.. ... ... ... .. [560]

#AY883833 ... ... A.. ... ... ... .. [560]

#AY883831 ... ... A.. ... ... ... .. [560]

#KY863454 ... ... A.. ... ... ... .. [560]

#EF208912 ... ... A.. ... ... ... .. [560]

#FJ526438 ... ... A.. ... ... ... .. [560]

#FJ526437 ... ... A.. ... ... ... .. [560]

#AF417749 ... ... A.. ... ... ... .. [560]

#JX139608 ... C.. A.. ... ... ... .. [560]
