# Supplementary material for: Identification of a biological form in the Anopheles stephensi laboratory colony using the odorant-binding protein 1 intron I sequence
Source: PLoS One. 2022 Feb 22;17(2):e0263836. doi: 10.1371/journal.pone.0263836 (PMC8863247; doi:10.1371/journal.pone.0263836)
Supplement: S3 Fig — Bootstrap values >70 shown at nodes. (DOCX) [file pone.0263836.s003.docx]

#MZ269267 CAAGTTATCT CAACACTCTA ACCAAACTGA CCGTCCTGAC CCCATCATAG GGGGGTAGGC [ 60]

#MZ269268 .......... .......... .......... .......... .......... .......... [ 60]

#MZ269269 .......... .......... .......... .......... .......... .......... [ 60]

#MZ269270 .......... .......... .......... .......... .......... .......... [ 60]

#MZ269271 .......... .......... .......... .......... .......... .......... [ 60]

#MW017363 .......... .......... .......... .......... .......... .......... [ 60]

#MW017364 .......... .T......C. .......... .......... .......... .......... [ 60]

#KM052589 .......... .......... .......... .......... .......... .......... [ 60]

#KM052590 .......... .......... .......... .......... .......... .......... [ 60]

#AY702486 .......... .......... .......... .......... .......... .......... [ 60]

#AY702485 .......... .......... .......... .......... .......... .......... [ 60]

#AY702484 .......... .......... .......... .......... .......... .......... [ 60]

#AY702483 .......... .......... .......... .......... .......... .......... [ 60]

#AY702482 .......... .......... .......... .......... .......... .......... [ 60]

#AY365050 .......... .......... .......... .......... .......... .......... [ 60]

#AY365049 .......... .......... .......... .......... .......... .......... [ 60]

#AY702490 .......... .......... .......... .......... .......... .......... [ 60]

#AY157316 .......... .......... .......... .......... .......... .......... [ 60]

#AY157678 .......... .......... .......... .......... .......... .......... [ 60]

#EU359681 .......... .......... .......... .......... .......... .......... [ 60]

#EU359680 .......... .......... .......... .......... .......... .......... [ 60]

#EU359679 .......... .......... .......... .......... .......... .......... [ 60]

#EU359678 .......... .......... .......... .......... .......... .......... [ 60]

#EU359677 .......... .......... .......... .......... .......... .......... [ 60]

#EU359676 .......... .......... .......... .......... .......... .......... [ 60]

#EU359675 .......... .......... .......... .......... .......... .......... [ 60]

#EU359674 .......... .......... .......... .......... .......... .......... [ 60]

#EU359673 .......... .......... .......... .......... .......... .......... [ 60]

#EU359672 .......... .......... .......... .......... .......... .......... [ 60]

#EU359671 .......... .......... .......... .......... .......... .......... [ 60]

#EU359670 .......... .......... .......... .......... .......... .......... [ 60]

#EU359669 .......... .......... .......... .......... .......... .......... [ 60]

#EU359668 .......... .......... .......... .......... .......... .......... [ 60]

#EU359667 .......... .......... .......... .......... .......... .......... [ 60]

#EU359666 .......... .......... .......... .......... .......... .......... [ 60]

#EU359665 .......... .......... .......... .......... .......... .......... [ 60]

#EU359664 .......... .......... .......... .......... .......... .......... [ 60]

#EU359663 .......... .......... .......... .......... .......... .......... [ 60]

#EU359662 .......... .......... .......... .......... .......... .......... [ 60]

#EU359661 .......... .......... .......... .......... .......... .......... [ 60]

#EU346653 .......... .......... .......... .......... .......... .......... [ 60]

#EU346652 .......... .......... .......... .......... .......... .......... [ 60]

#DQ662409 .......... .......... .......... .......... .......... .......... [ 60]

#HQ703001 .......... .......... .......... .......... .......... .......... [ 60]

#MZ269267 TGTCGCAGCA TGGCGTGCTC GGATCCGCAT CCTTGTCGGG ACCGTGGGCG CTGAAAGTGA [120]

#MZ269268 .......... .......... .......... .......... .......... .......... [120]

#MZ269269 .......... .......... .......... .......... .......... .......... [120]

#MZ269270 .......... .......... .......... .......... .......... .......... [120]

#MZ269271 .......... .......... .......... .......... .......... .......... [120]

#MW017363 .......... .......... .......... .......... .......... .........G [120]

#MW017364 .......... .......... .......... .......... .......... .......... [120]

#KM052589 .......... .......... .......... .......... .......... .......... [120]

#KM052590 .......... .......... .......... .......... .......... .......... [120]

#AY702486 .......... .......... .......... .......... .......... .......... [120]

#AY702485 .......... .......... .......... .......... .......... .......... [120]

#AY702484 .......... .......... .......... .......... .......... .......... [120]

#AY702483 .......... .......... .......... .......... .......... .......... [120]

#AY702482 .......... .......... .......... .......... .......... .......... [120]

#AY365050 .......... .......... .......... .......... .......... .......... [120]

#AY365049 .......... .......... .......... .......... .......... .......... [120]

#AY702490 .......... .......... .......... .......... .......... .......... [120]

#AY157316 .......... .......... .......... .......... .......... .......... [120]

#AY157678 .......... .......... .......... .......... .......... .......... [120]

#EU359681 .......... .......... .......... .......... .......... .......... [120]

#EU359680 .......... .......... .......... .......... .......... .......... [120]

#EU359679 .......... .......... .......... .......... .......... .......... [120]

#EU359678 .......... .......... .......... .......... .......... .......... [120]

#EU359677 .......... .......... .......... .......... .......... .......... [120]

#EU359676 .......... .......... .......... .......... .......... .......... [120]

#EU359675 .......... .......... .......... .......... .......... .......... [120]

#EU359674 .......... .......... .......... .......... .......... .......... [120]

#EU359673 .......... .......... .......... .......... .......... .......... [120]

#EU359672 .......... .......... .......... .......... .......... .......... [120]

#EU359671 .......... .......... .......... .......... .......... .......... [120]

#EU359670 .......... .......... .......... .......... .......... .......... [120]

#EU359669 .......... .......... .......... .......... .......... .......... [120]

#EU359668 .......... .......... .......... .......... .......... .......... [120]

#EU359667 .......... .......... .......... .......... .......... .......... [120]

#EU359666 .......... .......... .......... .......... .......... .......... [120]

#EU359665 .......... .......... .......... .......... .......... .......... [120]

#EU359664 .......... .......... .......... .......... .......... .......... [120]

#EU359663 .......... .......... .......... .......... .......... .......... [120]

#EU359662 .......... .......... .......... .......... .......... .......... [120]

#EU359661 .......... .......... .......... .......... .......... .......... [120]

#EU346653 .......... .......... .......... .......... .......... .......... [120]

#EU346652 .......... .......... .......... .......... .......... .......... [120]

#DQ662409 .......... .......... .......... .......... .......... .......... [120]

#HQ703001 .......... .......... .......... .......... .......... .......... [120]

#MZ269267 GAGTGCTAAC ACAGAGAGAC ATACGAGGTA TGGTACACAA ACCTAAACAC ACACACACAC [180]

#MZ269268 .......... .......... .......... .......... .......... .......... [180]

#MZ269269 .......... .......... .......... .......... .......... .......... [180]

#MZ269270 .......... .......... .......... .......... .......... .......... [180]

#MZ269271 .......... .......... .......... .......... .......... .......... [180]

#MW017363 .......... .......... .......... .......... .......... .......... [180]

#MW017364 .......... .......... .......... .......... .......... .......... [180]

#KM052589 .......... .......... .......... .......... .......... .......... [180]

#KM052590 .......... .......... .......... .......... .......... .......... [180]

#AY702486 .......... .......... .......... .......... .......... .......... [180]

#AY702485 .......... .......... .......... .......... .......... .......... [180]

#AY702484 .......... .......... .......... .......... .......... .......... [180]

#AY702483 .......... .......... .......... .......... .......... .......... [180]

#AY702482 .......... .......... .......... .......... .......... .......... [180]

#AY365050 .......... .......... .......... .......... .......... .......... [180]

#AY365049 .......... .......... .......... .......... .......... .......... [180]

#AY702490 .......... .......... .......... .......... .......... .......... [180]

#AY157316 .......... .......... .......... .......... .......... .......... [180]

#AY157678 .......... .......... .......... .......... .......... .......... [180]

#EU359681 .......... .......... .......... .......... .......... .......... [180]

#EU359680 .......... .......... .......... .......... .......... .......... [180]

#EU359679 .......... .......... .......... .......... .......... .......... [180]

#EU359678 .......... .......... .......... .......... .......... .......... [180]

#EU359677 .......... .......... .......... .......... .......... .......... [180]

#EU359676 .......... .......... .......... .......... .......... .......... [180]

#EU359675 .......... .......... .......... .......... .......... .......... [180]

#EU359674 .......... .......... .......... .......... .......... .......... [180]

#EU359673 .......... .......... .......... .......... .......... .......... [180]

#EU359672 .......... .......... .......... .......... .......... .......... [180]

#EU359671 .......... .......... .......... .......... .......... .......... [180]

#EU359670 .......... .......... .......... .......... .......... .......... [180]

#EU359669 .......... .......... .......... .......... .......... .......... [180]

#EU359668 .......... .......... .......... .......... .......... .......... [180]

#EU359667 .......... .......... .......... .......... .......... .......... [180]

#EU359666 .......... .......... .......... .......... .......... .......... [180]

#EU359665 .......... .......... .......... .......... .......... .......... [180]

#EU359664 .......... .......... .......... .......... .......... .......... [180]

#EU359663 .......... .......... .......... .......... .......... .......... [180]

#EU359662 .......... .......... .......... .......... .......... .......... [180]

#EU359661 .......... .......... .......... .......... .......... .......... [180]

#EU346653 .......... .......... .......... .......... .......... .......... [180]

#EU346652 .......... .......... .......... .......... .......... .......... [180]

#DQ662409 .......... .......... .......... .......... .......... .......... [180]

#HQ703001 .......... .......... .......... .......... .......... .........T [180]

#MZ269267 ATGTGAGCAT GG-GTGAAGA GCGAGCGCGC GTCAAGTCGC ACGGTTCGAC CTCTAGTATC [240]

#MZ269268 .......... ..-....... .......... .......... .......... .......... [240]

#MZ269269 .......... ..-....... .......... .......... .......... .......... [240]

#MZ269270 .......... ..-....... .......... .......... .......... .......... [240]

#MZ269271 .......... ..-....... .......... .......... .......... .......... [240]

#MW017363 .......... ..-....... .......... .......... .......... .......... [240]

#MW017364 .......... ..-....... .......... .......... .......... ..T....... [240]

#KM052589 .......... ..-....... .......... .......... .......... .......... [240]

#KM052590 .......... ..-....... .......... .......... .......... .......... [240]

#AY702486 .......... ..-....... .......... .......... .......... .......... [240]

#AY702485 .......... ..A....... A......... .......... .......... .......... [240]

#AY702484 .......... ..-....... .......... .......... .......... .......... [240]

#AY702483 .......... ..-....... .......... .......... .......... .......... [240]

#AY702482 .......... ..-....... .......... .......... .......... .......... [240]

#AY365050 .......... ..-....... .......... .......... .......... .......... [240]

#AY365049 .......... ..-....... .......... .......... .......... .......... [240]

#AY702490 .......... ..-....... .......... .......... .......... .......... [240]

#AY157316 .......... ..-....... .......... .......... .......... .......... [240]

#AY157678 .......... ..-....... .......... .......... .......... .......... [240]

#EU359681 .......... ..-....... .......... .......... .......... .......... [240]

#EU359680 .......... ..-....... .......... .......... .......... .......... [240]

#EU359679 .......... ..-....... .......... .......... .......... .......... [240]

#EU359678 .......... ..-....... .......... .......... .......... .......... [240]

#EU359677 .......... ..-....... .......... .......... .......... .......... [240]

#EU359676 .......... ..-....... .......... .......... .......... .......... [240]

#EU359675 .......... ..-....... .......... .......... .......... .......... [240]

#EU359674 .......... ..-....... .......... .......... .......... .......... [240]

#EU359673 .......... ..-....... .......... .......... .......... .......... [240]

#EU359672 .......... ..-....... .......... .......... .......... .......... [240]

#EU359671 .......... ..-....... .......... .......... .......... .......... [240]

#EU359670 .......... ..-....... .......... .......... .......... .......... [240]

#EU359669 .......... ..-....... .......... .......... .......... .......... [240]

#EU359668 .......... ..-....... .......... .......... .......... .......... [240]

#EU359667 .......... ..-....... .......... .......... .......... .......... [240]

#EU359666 .......... ..-....... .......... .......... .......... .......... [240]

#EU359665 .......... ..-....... .......... .......... .......... .......... [240]

#EU359664 .......... ..-....... .......... .......... .......... .......... [240]

#EU359663 .......... ..-....... .......... .......... .......... .......... [240]

#EU359662 .......... ..-....... .......... .......... .......... .......... [240]

#EU359661 .......... ..-....... .......... .......... .......... .......... [240]

#EU346653 .......... ..-....... .......... .......... .......... .......... [240]

#EU346652 .......... ..-....... .......... .......... .......... .......... [240]

#DQ662409 .......... ..-....... .......... .......... .......... .......... [240]

#HQ703001 G......TG. ..-.A..... .A........ ........A. .G..C.AC.. .....A.... [240]

#MZ269267 AACCAACGGA TGTATCCACC ACAGCATATA AGGTTATCAC CAATTGCACG GGGACTTCCA [300]

#MZ269268 .......... .......... .......... .......... .......... .......... [300]

#MZ269269 .......... .......... .......... .......... .......... .......... [300]

#MZ269270 .......... .......... .......... .......... .......... .......... [300]

#MZ269271 .......... .......... .......... .......... .......... .......... [300]

#MW017363 .......... .......... .......... .......... .......... .......... [300]

#MW017364 .......... .......... .......... .......... .......... .......... [300]

#KM052589 .......... .......... .......... .......... .......... .......... [300]

#KM052590 .......... .......... .......... .......... .......... .......... [300]

#AY702486 .......... .......... .......... .......... .......... .......... [300]

#AY702485 .......... .......... .......... .......... .......... .......... [300]

#AY702484 .......... .......... .......... .......... .......... .......... [300]

#AY702483 .......... .......... .......... .......... .......... .......... [300]

#AY702482 .......... .......... .......... .......... .......... .......... [300]

#AY365050 .......... .......... .......... .......... .......... .......... [300]

#AY365049 .......... .......... .......... .......... .......... .......... [300]

#AY702490 .......... .......... .......... .......... .......... .......... [300]

#AY157316 .......... .......... .......... .......... .......... .......... [300]

#AY157678 .......... .......... .......... .......... .......... .......... [300]

#EU359681 .......... .......... .......... .......... .......... .......... [300]

#EU359680 .......... .......... .......... .......... .......... .......... [300]

#EU359679 .......... .......... .......... .......... .......... .......... [300]

#EU359678 .......... .......... .......... .......... .......... .......... [300]

#EU359677 .......... .......... .......... .......... .......... .......... [300]

#EU359676 .......... .......... .......... .......... .......... .......... [300]

#EU359675 .......... .......... .......... .......... .......... .......... [300]

#EU359674 .......... .......... .......... .......... .......... .......... [300]

#EU359673 .......... .......... .......... .......... .......... .......... [300]

#EU359672 .......... .......... .......... .......... .......... .......... [300]

#EU359671 .......... .......... .......... .......... .......... .......... [300]

#EU359670 .......... .......... .......... .......... .......... .......... [300]

#EU359669 .......... .......... .......... .......... .......... .......... [300]

#EU359668 .......... .......... .......... .......... .......... .......... [300]

#EU359667 .......... .......... .......... .......... .......... .......... [300]

#EU359666 .......... .......... .......... .......... .......... .......... [300]

#EU359665 .......... .......... .......... .......... .......... .......... [300]

#EU359664 .......... .......... .......... .......... .......... .......... [300]

#EU359663 .......... .......... .......... .......... .......... .......... [300]

#EU359662 .......... .......... .......... .......... .......... .......... [300]

#EU359661 .......... .......... .......... .......... .......... .......... [300]

#EU346653 .......... .......... .......... .......... .......... .......... [300]

#EU346652 .......... .......... .......... .......... .......... .......... [300]

#DQ662409 .......... .......... .......... .......... .......... .......... [300]

#HQ703001 CC..C.GAT. .......C.. ...AT..... G..A...... .TTG...GG. .AC......C [300]

#MZ269267 CCGGTTGGCT CGGGTCGAGT AACACTTGCG GCCCAACGCG CTCGTATCTT TCCTCGCATC [360]

#MZ269268 .......... .......... .......... .......... .......... .......... [360]

#MZ269269 .......... .......... .......... .......... .......... .......... [360]

#MZ269270 .......... .......... .......... .......... .......... .......... [360]

#MZ269271 .......... .......... .......... .......... .......... .......... [360]

#MW017363 .......... .......... .......... .......... .......... .......... [360]

#MW017364 .......... .......... .......... .......... .......... .......... [360]

#KM052589 .......... .......... .......... .......... .......... .......... [360]

#KM052590 .......... .......... .......... .......... .......... .......... [360]

#AY702486 .......... .......... .......... .......... .......... .......... [360]

#AY702485 .......... .......... .......... .......... .......... .......... [360]

#AY702484 .......... .......... .......... .......... .......... .......... [360]

#AY702483 .......... .......... .......... .......... .......... .......... [360]

#AY702482 .......... .......... .......... .......... .......... .......... [360]

#AY365050 .......... .......... .......... .......... .......... .......... [360]

#AY365049 .......... .......... .......... .......... .......... .......... [360]

#AY702490 .......... .......... .......... .......... .......... .......... [360]

#AY157316 .......... .......... .......... .......... .......... .......... [360]

#AY157678 .......... .......... .......... .......... .......... .......... [360]

#EU359681 .......... .......... .......... .......... .......... .......... [360]

#EU359680 .......... .......... .......... .......... .......... .......... [360]

#EU359679 .......... .......... .......... .......... .......... .......... [360]

#EU359678 .......... .......... .......... .......... .......... .......... [360]

#EU359677 .......... .......... .......... .......... .......... .......... [360]

#EU359676 .......... .......... .......... .......... .......... .......... [360]

#EU359675 .......... .......... .......... .......... .......... .......... [360]

#EU359674 .......... .......... .......... .......... .......... .......... [360]

#EU359673 .......... .......... .......... .......... .......... .......... [360]

#EU359672 .......... .......... .......... .......... .......... .......... [360]

#EU359671 .......... .......... .......... .......... .......... .......... [360]

#EU359670 .......... .......... .......... .......... .......... .......... [360]

#EU359669 .......... .......... .......... .......... .......... .......... [360]

#EU359668 .......... .......... .......... .......... .......... .......... [360]

#EU359667 .......... .......... .......... .......... .......... .......... [360]

#EU359666 .......... .......... .......... .......... .......... .......... [360]

#EU359665 .......... .......... .......... .......... .......... .......... [360]

#EU359664 .......... .......... .......... .......... .......... .......... [360]

#EU359663 .......... .......... .......... .......... .......... .......... [360]

#EU359662 .......... .......... .......... .......... .......... .......... [360]

#EU359661 .......... .......... .......... .......... .......... .......... [360]

#EU346653 .......... .......... .......... .......... .......... .......... [360]

#EU346652 .......... .......... .......... .......... .......... .......... [360]

#DQ662409 .......... .......... .......... .......... .......... .......... [360]

#HQ703001 ....G..T.. .....A...A ....TG.... .........T ...A...T.. .....AT... [360]

#MZ269267 CAGTTGCAGT CGCGAGACGT GCTCACGCCG TGTGGGTGAG TGAGGTTAGC ACGA-CAGGG [420]

#MZ269268 .......... .......... .......... .......... .......... ....-..... [420]

#MZ269269 .......... .......... .......... .......... .......... ....-..... [420]

#MZ269270 .......... .......... .......... .......... .......... ....-..... [420]

#MZ269271 .......... .......... .......... .......... .......... ....-..... [420]

#MW017363 .......... .......... .......... .........A .......... ....-..... [420]

#MW017364 .......... .......... .......... ....A..... .......... ....-..... [420]

#KM052589 .......... .......... .......... .......... .......... ....-..... [420]

#KM052590 .......... .......... .......... .......... .......... ....-..... [420]

#AY702486 .......... .......... .......... .......... .......... ....-..... [420]

#AY702485 .......... .......... .......... .......... .......... ....-..... [420]

#AY702484 .......... .......... .......... .......... .......... ....-..... [420]

#AY702483 .......... .......... .......... .......... .......... ....A..... [420]

#AY702482 .......... .......... .......... .......... .......... ....-..... [420]

#AY365050 .......... .......... .......... .......... .......... ....-..... [420]

#AY365049 .......... .......... .......... .......... .......... ....-..... [420]

#AY702490 .......... .......... .......... .......... .......... ....-..... [420]

#AY157316 .......... .......... .......... .......... .......... ....-..... [420]

#AY157678 .......... .......... .......... .......... .......... ....-..... [420]

#EU359681 .......... .......... .......... .......... .......... ....-..... [420]

#EU359680 .......... .......... .......... .......... .......... ....-..... [420]

#EU359679 .......... .......... .......... .......... .......... ....-..... [420]

#EU359678 .......... .......... .......... .......... .......... ....-..... [420]

#EU359677 .......... .......... .......... .......... .......... ....-..... [420]

#EU359676 .......... .......... .......... .......... .......... ....-..... [420]

#EU359675 .......... .......... .......... .......... .......... ....-..... [420]

#EU359674 .......... .......... .......... .......... .......... ....-..... [420]

#EU359673 .......... .......... .......... .......... .......... ....-..... [420]

#EU359672 .......... .......... .......... .......... .......... ....-..... [420]

#EU359671 .......... .......... .......... .......... .......... ....-..... [420]

#EU359670 .......... .......... .......... .......... .......... ....-..... [420]

#EU359669 .......... .......... .......... .......... .......... ....-..... [420]

#EU359668 .......... .......... .......... .......... .......... ....-..... [420]

#EU359667 .......... .......... .......... .......... .......... ....-..... [420]

#EU359666 .......... .......... .......... .......... .......... ....-..... [420]

#EU359665 .......... .......... .......... .......... .......... ....-..... [420]

#EU359664 .......... .......... .......... .......... .......... ....-..... [420]

#EU359663 .......... .......... .......... .......... .......... ....-..... [420]

#EU359662 .......... .......... .......... .......... .......... ....-..... [420]

#EU359661 .......... .......... .......... .......... .......... ....-..... [420]

#EU346653 .......... .......... .......... .......... ........C. ....-..... [420]

#EU346652 .......... .......... .......... .......... .......... ....-..... [420]

#DQ662409 .........A .......... .......... .......... .......... ....-..... [420]

#HQ703001 .TTG....C. ..A....T.. .......... ....T.A... A..T.A..A. .AC.-....T [420]

#MZ269267 GTGATTTATC ACCGCTTCTC CCGTCGCAT- CATTGTGACA GTG-GAGTCT [470]

#MZ269268 .......... .......... .........- .......... ...-...... [470]

#MZ269269 .......... .......... .........- .......... ...-...... [470]

#MZ269270 .......... .......... .........- .......... ...-...... [470]

#MZ269271 .......... .......... .........- .......... ...-...... [470]

#MW017363 .......... .......... .........- .......... ...-...... [470]

#MW017364 .......... .......... .........- .......... ...-...... [470]

#KM052589 .......... .......... .........- .......... ...-...... [470]

#KM052590 .......... .......... .........- .......... ...-...... [470]

#AY702486 .......... .......... .........- .......... ...-...... [470]

#AY702485 .......... .......... .........A .......... ...-...... [470]

#AY702484 .......... .......... .........- .......... ...-...... [470]

#AY702483 .......... .......... .........- .......... ...-...... [470]

#AY702482 .......... .......... .....A...- .......... ...-...... [470]

#AY365050 .......... .......... .........- .......... ...-...... [470]

#AY365049 .......... .......... .........- .......... ...-...... [470]

#AY702490 .......... .......... .........- .......... ...-...... [470]

#AY157316 .......... .......... .........- .......... ...-...... [470]

#AY157678 .......... .......... .........- .......... ...-...... [470]

#EU359681 .......... .......... .........- .......... ...-...... [470]

#EU359680 .......... .......... .........- .......... ...-...... [470]

#EU359679 .......... .......... .........- .......... ...-...... [470]

#EU359678 .......... .......... .........- .......... ...-...... [470]

#EU359677 .......... .......... .........- .......... ...-...... [470]

#EU359676 .......... .......... .........- .......... ...-...... [470]

#EU359675 .......... .......... .........- .......... ...-...... [470]

#EU359674 .......... .......... .........- .......... ...-...... [470]

#EU359673 .......... .......... .........- .......... ...-...... [470]

#EU359672 .......... .......... .........- .......... ...-...... [470]

#EU359671 .......... .......... .........- .......... ...-...... [470]

#EU359670 .......... .......... .........- .......... ...-...... [470]

#EU359669 .......... .......... .........- .......... ...-...... [470]

#EU359668 .......... .......... .........- .......... ...-...... [470]

#EU359667 .......... .......... .........- .......... ...-...... [470]

#EU359666 .......... .......... .........- .......... ...-...... [470]

#EU359665 .......... .......... .........- .......... ...-...... [470]

#EU359664 .......... .......... .........- .......... ...-...... [470]

#EU359663 .......... .......... .........- .......... ...-...... [470]

#EU359662 .......... .......... .........- .......... ...-...... [470]

#EU359661 .......... .......... .........- .......... ...-...... [470]

#EU346653 .......... .......... .........- .......... ...-...... [470]

#EU346652 .......... .......... .........- .......... ...A...... [470]

#DQ662409 .......... .......... .........- .......... ...-...... [470]

#HQ703001 .A...A.... .G...C.... GTC..A...- .TG..A.... .GA-..C... [470]
