# Supplementary material for: Identification of a biological form in the Anopheles stephensi laboratory colony using the odorant-binding protein 1 intron I sequence
Source: PLoS One. 2022 Feb 22;17(2):e0263836. doi: 10.1371/journal.pone.0263836 (PMC8863247; doi:10.1371/journal.pone.0263836)
Supplement: S1 Table — (DOCX) [file pone.0263836.s004.docx]

| Mysorensis biolocal form | **COI (758 bp)** | | **COII (560bp)** | | **ITS2 (470bp)** | | ***Anste*Obp1 (120 bp)** | |
| --- | --- | --- | --- | --- | --- | --- | --- | --- |
|  | **GenBank accessions no.** | **The closest available public sequence** | **GenBank accessions numbers** | **The closest available public sequence** | **GenBank accessions numbers** | **The closest available public sequence** | **GenBank accessions numbers** | **The closest available public sequence** |
|  | MW012492  MZ269698  MZ269699  MZ269700  MZ269701  MZ269702  MZ269703  MZ269704  MZ269705 | KT899888  AY877426  AY877428  AY877429  AY877427  AF417713  KR817728 | MW431057  MZ420723  MZ420724  MZ420725  MZ420726  MZ420727  MZ420728  MZ420729  MZ420730 | KT899888  AF425844  DQ022847  DQ022846  DQ022845  DQ026675  AY883832  AY883830  AY883837  AY883836  AY883835  AY883834  AY883833  AY883831  KY863454  EF208912  FJ526438  FJ526437  AF417749  JX139608 | MW017363  MW017364  MZ269267  MZ269268  MZ269269  MZ269270  MZ269271 | KM052589  KM052590  AY702486  AY702485  AY702484  AY702483  AY702482  AY365050  AY365049  AY702490  AY157316  AY157678  EU359681  EU359680  EU359679  EU359678  EU359677  EU359676  EU359675  EU359674  EU359673  EU359672  EU359671  EU359670  EU359669  EU359668  EU359667  EU359666  EU359665  EU359664  EU359663  EU359662  EU359661  EU346653  EU346652  DQ662409  HQ703001 | MW013512  MW013513  MW013514  MW013515  MW013516  MW013517  MW013518  MW013519  MW013520  MZ420719  MZ420720  MZ420721  MZ420722 | 1. KJ557463  *An. stephensi* A (type)  2. KJ557452  *An*. *stephensi* B (intermediate)  3. KJ557455  *An. stephensi* C (mysorensis) |
