# Supplementary material for: Identification of a biological form in the Anopheles stephensi laboratory colony using the odorant-binding protein 1 intron I sequence
Source: PLoS One. 2022 Feb 22;17(2):e0263836. doi: 10.1371/journal.pone.0263836 (PMC8863247; doi:10.1371/journal.pone.0263836)
Supplement: Attachment — Submitted filename: PONE-D-21-36790.pdf [file pone.0263836.s005.pdf]

PLOS ONE

Speculation on the possibility for introducing Anopheles stephensi as a species complex: secondary evidence based on odorant-binding protein 1 intron I sequence

--Manuscript Draft--

Manuscript Number:	PONE-D-21-36790
Article Type:	Research Article
Full Title:	Speculation on the possibility for introducing Anopheles stephensi as a species complex: secondary evidence based on odorant-binding protein 1 intron I sequence
Short Title:	Species complexes of Anopheles stephensi
Corresponding Author:	Jehangir Khan, MS<br>Abdul Wali Khan University Mardan (AWKUM), Khyber Pakhtunkhwa, Pakistan<br>Mardan, KPK PAKISTAN
Keywords:	
Abstract:	Background<br>Anopheles stephensi Listen (1901) is a major vector of malaria in Asia and has recently been found in some regions of Africa. An. stepehnsi species complex is suspected to have three sibling species: type, intermediate, and mysorensis, each with its own vector competence (malaria) and ecology. To identify the species complex of our An. stephensi insectary colony, we used the morphological features of eggs and genetic markers such as Anste Obp1 (Anopheles stephensi odorant binding protein 1), mitochondrial oxidases subunit 1 and 2 (COI and COII), and nuclear internal transcribed spacer 2 locus (ITS2).<br>Methods<br>Eggs were collected from individual mosquito (n = 50) and counted for the number of ridges under stereomicroscope. Genomic DNA was extracted from female mosquitoes. After the amplification of partial fragments of Anste Obp1, COI, COII and ITS2 genes, the PCR products were purified and sequenced. Phylogenetic analysis was performed after aligning query sequences against the submitted sequences in GenBank using MEGA vx.<br>Results<br>The range of ridges number on each egg float was 12-13 that corresponds to the mysorensis form of An. stephensi . The generated COI, COII and ITS2 sequences showed 100%, 99.46% and 99.29% similarity with the sequences deposited for Chinese, Indian and Iranian strains of An. stephensi , respectively. All the generated Anste Obp1 intron I region sequences matched 100% with the sequences deposited for An. stephensi sibling C (mysorensis form) from Iran and Afghanistan.<br><br>Conclusions<br>This manuscript precisely describes the morphological and molecular details of the 'var mysorensis' form of An. stephensi that could be exploited in elucidating its classification as well as in differentiation from other biotypes of the same or other anopheline species. Based on our findings, we recommend AnsteObp 1 as a robust genetic marker for rapid and accurate discrimination (taxonomic identification) of the An. stephensi species complex, rather than the COI, COII, and ITS2 marker, which could only be utilized for interspecies (Anopheles) differentiation.
Order of Authors:	Jehangir Khan, MS
	Saber Gholizadeh, PhD
	Dongjing Zhang
	Wang Gang
	Yu Wu
	Yan Guo
	Xiaoying Zheng
	Wu Zhongdao

Powered by Editorial Manager® and ProduXion Manager® from Aries Systems Corporation

Additional Information:	
**Question**	**Response**
**Financial Disclosure**<br><br>Enter a financial disclosure statement that describes the sources of funding for the work included in this submission. Review the submission guidelines for detailed requirements. View published research articles from *PLOS ONE* for specific examples.<br><br>This statement is required for submission and **will appear in the published article** if the submission is accepted. Please make sure it is accurate.<br><br>**Unfunded studies**<br>Enter: *The author(s) received no specific funding for this work.*<br><br>**Funded studies**<br>Enter a statement with the following details:<br>• Initials of the authors who received each award<br>• Grant numbers awarded to each author<br>• The full name of each funder<br>• URL of each funder website<br>• Did the sponsors or funders play any role in the study design, data collection and analysis, decision to publish, or preparation of the manuscript?<br>• **NO** - Include this sentence at the end of your statement: *The funders had no role in study design, data collection and analysis, decision to publish, or preparation of the manuscript.*<br>• **YES** - Specify the role(s) played.<br><br>* typeset	This study is supported by the National Research and Development Plan of China (No. 2016YFC1200500) and 111 project (B12003).<br>Prof Wu Zhongdao has received this funding.
**Competing Interests**<br><br>Use the instructions below to enter a competing interest statement for this submission. On behalf of all authors, disclose any competing interests that could be perceived to bias this work—acknowledging all financial support and any other relevant financial or non-financial competing interests.	The authors have declared that no competing interests exist.

Powered by Editorial Manager® and ProduXion Manager® from Aries Systems Corporation

This statement is required for submission and **will appear in the published article** if the submission is accepted. Please make sure it is accurate and that any funding sources listed in your Funding Information later in the submission form are also declared in your Financial Disclosure statement.<br><br>View published research articles from *PLOS ONE* for specific examples.<br><br>**NO authors have competing interests**<br><br>Enter: *The authors have declared that no competing interests exist.*<br><br>**Authors with competing interests**<br><br>Enter competing interest details beginning with this statement:<br><br>*I have read the journal's policy and the authors of this manuscript have the following competing interests: [insert competing interests here]*<br><br>* typeset	
**Ethics Statement**<br><br>Enter an ethics statement for this submission. This statement is required if the study involved:<br><br>• Human participants<br>• Human specimens or tissue<br>• Vertebrate animals or cephalopods<br>• Vertebrate embryos or tissues<br>• Field research<br><br>Write "N/A" if the submission does not require an ethics statement.<br><br>General guidance is provided below. Consult the submission guidelines for detailed instructions. **Make sure that all information entered here is included in the**	N/A

Methods section of the manuscript.<br><br>**Format for specific study types**<br><br>**Human Subject Research (involving human participants and/or tissue)**<br>• Give the name of the institutional review board or ethics committee that approved the study<br>• Include the approval number and/or a statement indicating approval of this research<br>• Indicate the form of consent obtained (written/oral) or the reason that consent was not obtained (e.g. the data were analyzed anonymously)<br><br>**Animal Research (involving vertebrate animals, embryos or tissues)**<br>• Provide the name of the Institutional Animal Care and Use Committee (IACUC) or other relevant ethics board that reviewed the study protocol, and indicate whether they approved this research or granted a formal waiver of ethical approval<br>• Include an approval number if one was obtained<br>• If the study involved *non-human primates*, add *additional details* about animal welfare and steps taken to ameliorate suffering<br>• If anesthesia, euthanasia, or any kind of animal sacrifice is part of the study, include briefly which substances and/or methods were applied<br><br>**Field Research**<br><br>Include the following details if this study involves the collection of plant, animal, or other materials from a natural setting:<br>• Field permit number<br>• Name of the institution or relevant body that granted permission	
**Data Availability**<br><br>Authors are required to make all data underlying the findings described fully available, without restriction, and from the time of publication. PLOS allows rare exceptions to address legal and ethical concerns. See the PLOS Data Policy and FAQ for detailed information.	Yes - all data are fully available without restriction

Powered by Editorial Manager® and ProduXion Manager® from Aries Systems Corporation

A Data Availability Statement describing where the data can be found is required at submission. Your answers to this question constitute the Data Availability Statement and **will be published in the article**, if accepted.<br><br>**Important:** Stating 'data available on request from the author' is not sufficient. If your data are only available upon request, select 'No' for the first question and explain your exceptional situation in the text box.<br><br>Do the authors confirm that all data underlying the findings described in their manuscript are fully available without restriction?	
**Describe where the data may be found in full sentences. If you are copying our sample text, replace any instances of XXX with the appropriate details.**<br><br>• If the data are **held or will be held in a public repository**, include URLs, accession numbers or DOIs. If this information will only be available after acceptance, indicate this by ticking the box below. For example: *All XXX files are available from the XXX database (accession number(s) XXX, XXX.*).<br>• If the data are all contained **within the manuscript and/or Supporting Information files**, enter the following: *All relevant data are within the manuscript and its Supporting Information files.*<br>• If neither of these applies but you are able to provide **details of access elsewhere**, with or without limitations, please do so. For example:<br><br>*Data cannot be shared publicly because of [XXX]. Data are available from the XXX Institutional Data Access / Ethics Committee (contact via XXX) for researchers who meet the criteria for access to confidential data.*<br><br>*The data underlying the results*	All relevant data are within the manuscript and its Supporting Information files.

presented in the study are available from (include the name of the third party and contact information or URL).<br>• This text is appropriate if the data are owned by a third party and authors do not have permission to share the data.<br><br>* typeset	
Additional data availability information:	

Powered by Editorial Manager® and ProduXion Manager® from Aries Systems Corporation

Manuscript Click here to access/download;Manuscript;Manuscript.docx

Speculation on the possibility for introducing *Anopheles stephensi* as a species complex: secondary evidence based on odorant-binding protein 1 intron I sequence

Jehangir Khan [1,2,3], Saber Gholizadeh[4,5], Dongjing Zhang[2], Wang Gang[1],Yu Wu[1,2], Yan Guo[1,2], Xiaoying Zheng[1,2], Zhongdao Wu [1,6]*

[1]Department of Parasitology, Zhongshan School of Medicine, Sun Yat-sen University, Guangzhou, Guangdong, China.

[2]Sun Yat-sen University-Michigan State University Joint Center of Vector Control for Tropical Disease, Zhongshan School of Medicine, Guangzhou, Guangdong, China.

[3]Department of Zoology, Abdul Wali Khan University Mardan, Khyber Pakhtunkhwa, Pakistan.

[4]Cellular and Molecular Research Center, Urmia University of Medical Sciences, Urmia, Iran.

[5]Medical Entomology Department, School of Public Health, Urmia University of Medical Sciences, Urmia, Iran

[6]Key laboratory of Tropical Disease Control (Sun Yat-sen University), Ministry of Education, Guangzhou, Guangdong 510080, China.

E-mail addresses

Jehangir Khan: Abu_amna2013@hotmail.com

Saber Gholizadeh: sabergholizadeh@yahoo.com; saber@umsu.ac.ir

Dongjing Zhang: zhangdongjing06@163.com

Wang Gang: wangg69@mail.sysu.edu.cn

Yu Wu: wuyu@mail.sysu.edu.cn

Yan Guo: yanguo20916129@163.com

Xiaoying Zheng: zhengxy@mail.sysu.edu.cn

Wu Zhongdao: wuzhd@mail.sysu.edu.cn

Corresponding author: wuzhd@mail.sysu.edu.cn

Abstract

Background

Anopheles stephensi Listen (1901) is a major vector of malaria in Asia and has recently been found in some regions of Africa. *An. stepehnsi* species complex is suspected to have three sibling species: type, intermediate, and mysorensis, each with its own vector competence (malaria) and ecology. To identify the species complex of our *An. stephensi* insectary colony, we used the morphological features of eggs and genetic markers such as *Anste*Obp1 (*Anopheles stephensi* odorant binding protein 1), mitochondrial oxidases subunit 1 and 2 (COI and COII), and nuclear internal transcribed spacer 2 locus (ITS2).

Methods

Eggs were collected from individual mosquito (n = 50) and counted for the number of ridges under stereomicroscope. Genomic DNA was extracted from female mosquitoes. After the amplification of partial fragments of *Anste*Obp1, COI, COII and ITS2 genes, the PCR products were purified and sequenced. Phylogenetic analysis was performed after aligning query sequences against the submitted sequences in GenBank using MEGA vx.

Results

The range of ridges number on each egg float was 12-13 that corresponds to the mysorensis form of *An. stephensi*. The generated COI, COII and ITS2 sequences showed 100%, 99.46% and 99.29% similarity with the sequences deposited for Chinese, Indian and Iranian strains of *An. stephensi*, respectively. All the generated *Anste*Obp1 intron I region sequences matched 100% with the sequences deposited for *An. stephensi* sibling C (mysorensis form) from Iran and Afghanistan.

Conclusions

This manuscript precisely describes the morphological and molecular details of the 'var mysorensis' form of *An. stephensi* that could be exploited in elucidating its classification as well as in differentiation from other biotypes of the same or other *anopheline* species. Based on our findings, we recommend *AnsteObp*1 as a robust genetic marker for rapid and accurate discrimination (taxonomic identification) of the *An. stephensi* species complex, rather than the COI, COII, and ITS2 marker, which could only be utilized for interspecies (*Anopheles*) differentiation.

Key words: *Anopheles stephensi* sibling C, molecular genetic characterization, *AnteOBP1*, phylogenetic analysis.

Background

A wide variety of medically important insects belong to cryptic species complexes, which are morphologically identical (isomorphic), but reproductively isolated and have different seasonal prevalence, host preference, infection rates, resting habits, and biting cycles [1,2,3]. For instance, around 70 out of 482 species of anopheline mosquitoes act as vectors for malaria parasites and nearly 30 complexes of these have been identified so far in the world [4,5,6]. Because of the discovery of new biological species, the number of *Anopheles* vector species is rapidly rising [4,5,7,8]. The available information regarding the biology and distribution of *An. gambiae*, *An. culicifacies*, and *An. dirus* complexes in Africa, India, and Thailand, respectively, has demonstrated the importance of identifying the members of these species complexes [5]. Failure to discriminate among the vector and non-vector sibling species of anopheline species complexes may seriously mislead the malaria epidemiological mapping and the subsequent vector control strategies [5]. Lack of adequate knowledge about vector species complexes is playing a

significant role in the current worsening scenario of human malaria in the Asian-Pacific area [9], with worldwide malaria cases rising from 217 million in 2016 to 219 million in 2017 and around 229 million in 2019.

An. stephensi is one of the dominant malaria vectors in Middle East, the Indian subcontinent, Iran, Iraq, Bangladesh, south China, Myanmar, Thailand and Ethiopia [9,11,12]. Based on egg morphometric analysis, *An. stephensi* has three biological forms i.e. mysorensis, intermediate and type form [3] and these were identified as sibling species as Species C, Species B and Species A respectively [3,5]. The type biological form is an efficient vector of malaria in urban areas [13] whereas mysorensis is a poor vector (highly zoophilic) and limited only to rural areas, although it is susceptible to *Plasmodium vivax* (VK210B) [3,8,14]. The intermediate biological form is reported from rural and peri-urban areas with very little information about its vectorial capacity [6,15]. Despite efficient controlling strategies for malaria, *An. stephensi* is increasing in its geographic range. Thus, there is a dire need for the precise identification of members of the *Anopheles stephensi* and also for the members of the other *Anopheles* complexes which is crucial in malaria surveillance, effective control, and elimination strategies [5,15].

Information regarding population genetics of *An. stephensi* is still limited [16,17]. The mitochondrial oxidases subunit 1 and 2 (COI and COII), ribosomal internal transcribed spacer 2 (rDNA-ITS2) and domain-3 (D3) loci are the common molecular markers used, but none of them have distinguished accurately the biological forms of *An. stephensi* [15,17,18]. Alternatively, intron I sequences of Odorant-binding protein 1 has been recently shown to be potential genetic marker to differentiate members of the *An. stephensi* complex [15]. Accurate identification, the spatial distribution and population dynamics of cryptic species of the *An. stepehnsi* complex has

major human health implications since it directly impacts the vector control and disease management strategies [4,5].

Consequently, this study was designed with the following objectives: (i) to assess the potential of COI and COII, and ITS2 (routinely used markers) genes variations for reconstructing the phylogeny and recognition of cryptic species of *An. stephensi* in our insectary (ii) to demonstrate (as a secondary evidence) the *AnsteObp*1 intron I sequence a robust marker for rapid and accurate identification of *An. stephensi* and (iii) finally to introduce an optimized and easy protocol for sibling species identification of *An. stephensi*, based on the current molecular and morphological data.

It is indispensable to accurately characterize the insectary colony that could be used in vector control strategies such as *Wolbachia*-based, and Gene drive, etc. These developing technologies are becoming more popular and important for vector population replacement/suppression, but they are highly species-specific. The preliminary sequence data (associated with mysorensis) generated through this study may contribute well to the knowledge and reliable identification (taxonomic and phylogenetics) of the mysorensis form of *An. stephensi*.

Material and Methods

Colony maintenance

The colony of *An. stephensi* (Hor strain) has been maintained over 6 years in the insectary in 30×30×30 cm cages at Sun Yat-sen University, Guangzhou, Guangdong Province of China. Originally, this species was obtained with the courtesy of Wen-Yue Xu from Department of Pathogenic biology, Third Military Medical University, Chongqing China [19]. The rearing conditions were 28 ± 2 ℃, 70 ± 5 % RH, and a 12:12 (L: D) h photoperiod with a 10% (W/V)

sugar solution. Plastic trays (30 × 40 × 8 cm) were used for larvae rearing with deionized water and fed with IAEA 2 larval food in accordance with the standard explained procedure [20].

Mosquito feeding, collection and morphological study of eggs

After 5-7 days of adult emergence, the female mosquitoes were allowed to feed on anesthetized white mice (Kunming strain) for 30 minutes to start egg development. After blood feeding, about 50 engorged females were randomly isolated and kept in individual properly labeled plastic tubes (50 mL) (one mosquito/tube) with a dump paper at the bottom of each tube for eggs collection. The tubes were provided with cotton soaked in 10% sugar solution. After three days, the adult females were processed further for molecular analysis. About 50 eggs were mounted on slide (each time) with a drop of water and examined under stereomicroscope with 40 × (bright field illumination) magnification to count the number of ridges on eggs (one side) as described previously [3,16].

DNA extraction and PCR

After egg laying process, individual female mosquito from each tube was processed for DNA extraction using Dongsheng Biotech DNA extraction kit according to the manufacturer's instructions. Briefly, one mosquito was taken in 1.5 mL tube containing around 500 µL STE buffer and a small steel ball, and homogenized (50 Hzs for 30-60 seconds). Then 5 µL of this grinding solution (for each sample) was mixed with 18 µL of DNA extraction solution in a PCR tube, mixed well and incubated for 2 minutes at room temperature. Samples were processed for PCR with thermal condition at 95℃ for 10 minutes. Afterwards, 2 µL of neutralizing fluid was added to each PCR product, mixed well and incubated for several minutes at room temperature.

Finally, the extracted DNA was either kept at -20 ℃ or immediately processed for further amplification of target gene.

Amplification of COI, COII, ITS2, and *AnsteObp1* fragments

PCR was performed for individual mosquito (n = 50) to amplify COI, COII, ITS2 and *Anste*Obp1 partial genes. The PCR reactions were carried out in a 25 μL volume and the details of used primers and PCR conditions for each marker are presented in Table 1. Double distilled H_2O was used as negative control instead of template DNA in PCR reactions. The PCR products were purified by using TaKaRa agarose Gel DNA extraction Kit (Japan) and the amplicons were subsequently sequenced bi-directionally (both directions) using Sanger sequencing technology.

Sequence analysis and phylogenetic tree construction

The sequences were trimmed to remove any primer or other nucleotide contamination and double checked with Chromas software version 2.31 (www.technelysium.com.au/chromas.html). The final sequences were aligned using ClustalW [21] with the homologous sequences downloaded from GenBank and phylogenetic trees were constructed using distance Neighbor-joining and maximum likelihood Methods based on the Tamura–Nei model in MEGA7 [22].

A 120 bp fragment of *Anste*Obp1 intron I region from sequenced specimens selected from 845 bp sequenced region was used for analysis. *An. stephensi* sibling species sequences, A (KJ557463), B (KJ557452), C (KJ557455) [15] were used as representative for sequence comparisons and phylogenetic tree construction.

Results

Morphological analysis of the eggs

A total of 500 eggs were examined. We detected uniformity in all the observed eggs with ridges number 12-13 per egg [Fig. 1]. Based on the previously reported range of egg ridges, our laboratory mosquito colony was identified as the mysorensis biological form of *An. stephensi*. The previously defined criteria for identifying these three biological forms on the basis of ridges on floating eggs are about 10–15 (mysorensis), 15–17 (intermediate) and 17–22 egg ridges (type form) [23,3].

Molecular Sequence Analysis

Phylogenetic analysis of mitochondrial oxidase subunit I (COI)

Among 50 samples randomly selected for DNA extraction, 38 samples were sequenced; 9 samples for each of COI and COII, 7 for ITS2 while 13 samples were for *AsteObp1*. The size of sequenced COI (MW012492 and MZ269698-MZ269705) [Supplementary Table] region of the lab strain was 839 bp and trimmed to 758 bp with GenBank sequences and used for analysis and phylogenetic tree construction. There are 101 sequences of *An. stephensi* COI gene submitted to GenBank, of them, seven sequences (from Iran, India, China and Brazil) were compatible with our lab strain and included in the sequence analysis (Fig. 2 and supplementary fig 1). Multiple sequence alignment showed that the similarity between lab strain COI sequence and GenBank sequences was 99.87-100%. There were 7 mismatches in COI sequences as a transversion and 6 transitions (Fig. 2 and supplementary fig 1). Interestingly, a sequence directly submitted from China [24] was 100% similar to our lab strain COI sequence (Fig. 2). Interestingly, COI sequence of *An. stephensi* from Iran, India, Brazil and China distributed in three different clades in a phylogenetic tree (supplementary fig 1) (it will be because the sequences were from *An.*

stephensi sensu lato). Our lab strain sequence was placed with Chinese *An. stephensi* sequence in the same clade.

Phylogenetic analysis of mitochondrial oxidase subunit II (COII)

The COII sequences of *An. stephensi* (*n* = 24) extracted from GenBank were compared with our lab strain sequences (*n*=9). Four sequences were excluded because of shorter sequence size. COII sequences (560bp; MW431057, and MZ420723-MZ420730) of lab strain showed 99.82-100% similarity within nine sequences. A limited variation was because of two mismatches in 195 (C/T), 215 (A/G) and 523 (T/A) nucleotides as transition and transversion, respectively (Supplementary fig 2). The similarities between our sequences and 20 *An. stephensi* COII sequences available in GenBank deposited from different countries were 98.75-99.82% (Fig. 3 and supplementary fig 2). Less than 2% variation was because of 13 mismatches as transition (*n* = 11) and transversion (*n* = 2) (Fig. 3 and supplementary fig 2). Interestingly, mismatches in 195, 454, and 547 positions were specific to lab strain (Supplementary fig 2). Phylogenetic tree constructed based on *An. stephensi* COII sequences categorized the sequences in 3 clades (Fig. 3). Lab strain sequence was placed in a separate clade together with *An. stephensi* COII sequences from India and Iran (Fig 3).

Phylogenetic analysis of nuclear internal transcribed spacer 2 locus (ITS2)

After BLAST, *An. stephensi* rDNA-ITS2 sequences were extracted from GenBank (*n* = 118). These sequences were previously submitted from Iran, India, Iraq, Saudi Arabia and Sri Lanka. Eleven sequences from Sri Lanka were partial and therefore, excluded from the study. Finally, 37 representative rDNA-ITS2 sequences from GenBank and sequences obtained in the current study (n=7; MW017363 and MW017364, MZ269267- MZ269271) (470bp) were used for

analysis and phylogenetic tree construction (Fig. 4 and supplementary fig 3). Comparisons of our new lab strain sequences showed 98.71% similarity with each other, while they were randomly selected from the same colony. BLAST analysis of obtained sequences showed 97.63-100% similarities with sequences reported from Iran, India, Iraq and Saudi Arabia [Fig 4]. Interestingly, a sequence from India, HQ703001, showed 82.19-82.97% similarity with other rDNA-ITS2 sequences of *An. stephensi*, while its similarity with AY702482 from Iran was 98.28-99.79% (Fig. 4 and supplementary fig 3). The similarity among Lab sequences with others was 97.63-99.57% (Fig. 4). The topology of phylogenetic tree based on rDNA-ITS2 sequences of *An. stephensi* was similar to COI and COII having 3 clades with lower bootstrap values for clades (Supplementary fig. 3).

Phylogenetic analysis of *An. stephensi* odorant binding protein 1 (*Anste*Obp1)

Multiple sequence alignment showed 100% similarity of *Anste*Obp1 intron I sequence among thirteen *An. stephensi* specimens (MW013512-MW013520 and MZ420719-MZ420722). They were 100% similar with *An. stephensi* sibling C (mysorensis) (from Iran and Afghanistan), while their similarity with *An. stephensi* sibling A and B was 85% and 75.65%, respectively (Fig. 5). The *Anste*Obp1intron I sequences obtained in this study were clustered in a separate clade together with *An. stephensi* sibling C (mysorensis) in a based phylogenetic tree (Fig 6). As shown previously, [15, 16] *An. stephensi* sibling species A and B were placed in the separate clades (Fig. 6).

Discussion

Traditionally, *An. stephensi* was classified into two races based on the number of egg ridges [23], the '*mysorensis*' and '*type*', which were considered as sub-species [25,26] or sympatric species [27]. Later studies used different methods to give specific taxonomic status to these three

ecological/biological forms that differed in ridges number on the eggs, namely genetic crosses [3,38,16], cuticle hydrocarbons [29], chromosome karyotypes [30,31,32,33], spiracular index [37,38], differences in mating characteristics and behavior [5], cytogenetic properties [30,31], and molecular markers (ITS2, D3 loci, CO1 and COII) [18]. But none of these methods could clearly differentiate and give species status to the biological forms of *An. stephensi* [17, 18, 26]. Recently, *Anste*Obp1 is reported as a new marker for identification of the Asian main malaria vector, *An. stephensi*. In the current study, we assessed the effectiveness of commonly used (COI, COII, and ITS2) and novel (*Anste*Obp1) markers, as well as morphological features (egg ridge counts), in identifying the biological form of *An. stephensi*. The extensive morphological analysis of our mosquito eggs showed that ridges number in the range 12-13/egg corresponds to the mysorensis form of *An. stephensi* [3,23]. Our results are in accordance with the previously reported range of ridges number (mysorensis) i.e. 11 to 14 [Nagpal et al., 2003], 13-14 [Rao et al., 1938], 10-14 [Subbarao et al., 1987]. Similarly, the phylogenetic analysis (Fig. 6) of *Anste*Obp1 sequences of our mosquito strain showed 100% similarity with sibling species C reported from Iran and Afghanistan (the neighboring country of China). Although the current study has a limitation in that we only used the available lab strain of *An. stephensi* (wild mosquitoes were not available because of strict vector control measures in China), in a previous study [16], the entire wild collection of three strains of *An. stephensi* was successfully discriminated using *Anste*Obp1. This identification was attributed to the form specific/associated mutations (4-15%) within the intron region of *Anste*Obp1 between biological forms but no significant variation was noticed within the biological forms [16]. There was 99-100% similarity in the amino acids sequences of *Anste*Obp1among these members, with a single substitution (non-synonymous) in the type form [16]. Taking together, our mysorensis associated preliminary

sequence data may be exploited as representative/reference sequences for sequence comparisons and phylogenetic tree construction in similar studies in the future. Finally, our investigations, based on egg morphology and sequence analysis, endorse the use (independent) of the *Anste*Obp1 intron I sequence as a new molecular tool for quick and reliable identification of all the three biological forms of *An. stephensi*.

Vector control is fundamental for preventing the spread of malaria. Understanding population genetic structure of mosquito is imperative for shaping prevention strategies, particularly *Wolbachia*-based, Gene drive etc. The level of gene flow between mosquito populations can be predicted using population genetics research [Gakhar et al., 2013]. There are conflicts in the results regarding intra-species cross mating experiments. For example, reciprocal crosses between mysorensis and type strains reported a definite incompatibility [28]. Others demonstrated variations in the reproductive capacity within these biological forms of *An. stephensi* [34]. In contrast, no hybrid sterility was reported during a type-mysorensis cross experiments [27]. Subbarao et al (1987) did not find sterility in crossing experiments between the laboratory strains of the three ecological forms/biological forms [3]. As a result, these experiments provide perplexing results, which should be repeated after precise recognition of members of this species complex using effective genetic marker, such as AnsteObp1, and following the techniques outlined here [Table 1]. Further, there is a dire need to see whether pre-mating (reproductive isolation) barriers exist among these forms in the field. Accurate identification and exploring mating compatibility/incompatibility of the wild populations with the released (lab) strain is essential for the successful operation and field application of new emerging technologies i.e. *Wolbachia*-based that has been implemented for the suppression/replacement of wild *Aedes* mosquito population in a dozens of countries to control

dengue and Zika viruses [35]. If both the wild and lab (*Wolbachia* infected) mosquito populations are compatible, this approach could be used to eradicate malaria vectors in the wild.

Regarding the suitability of ITS2, COI and COII for distinguishing the biotypes of *An. stephensi*, our current observations and previous studies [17,18] confirm that these markers are not the suitable markers (based on high sequence similarity). In contrast, a new study reports both COI and COII (gene variation) as suitable markers to recognize the complexes of *An. gambiae* and *An. albitarsis* [36]. Yet our BLAST searches at GenBank database for either of ITS2, COI and COII sequences once again revealed them inappropriate for distinguishing our species strain. Our results are in accordance with [13,18,37,37]. Here, the phylogenetic analysis for COI, COII and ITS2 indicated our species 100%, 99.46% and 99.29% similar to other Chinese, Indian and Iranian strains of *An. stephensi* (Figs. 2, 3 and 4). Consequently, this indicates that aforesaid markers could be recommended only for identifying the species of *An. stephensi* (interspecies of *Anopheles* mosquito). Thus the independent use of *AnsteObp1* and associated protocols mentioned in this study are recommended to be used in future investigations that involve distinguishing the members of *An. stephensi* (intra-species variation).

Conclusion

This study finds *AnsteObp1* as a robust genetic marker for the identification of members of the *An. stephensi* complex. We support the hypothesis based on the inability of COI, COII, and ITS2 to identify *An. stephensi*'s sibling species. This study provides important information on morphological and molecular characterization of mysorensis biological form and the associated protocols. Conducting comprehensive entomological surveillance with precise identification of sibling species of *An. stephensi* complex contribute significantly to the ongoing malaria control

strategies. Consequently, we urge more research on the *An. stephensi* complex's bionomics, seasonal abundance, host and habitat preferences, plasmodium parasite susceptibility, biting cycle, and response to vector control strategies.

Declaration

Ethics approval and consent to participate

Not applicable

Consent for Publication

Not applicable

Availability of data and material

All the sequence data generated or analyzed during this study are submitted to NCBI and the assigned accession numbers are included in this manuscript.

Competing interests

The authors declare there are no competing interests.

Funding

This study is supported by the National Research and Development Plan of China (No. 2016YFC1200500) and 111 project (B12003).

Authors' contributions

Conceived and designed the experiments: ZW, JK, XZ, YW. Analyzed the data: SG, JK. Contributed reagents/materials/analysis tools: XZ, ZW, YW. Wrote the paper: JK. Critically revised the MS and provided suggestions and comments on the manuscript: SG, ZX, MZ, DZ. Figures formation: SG. Interpreted and adjusted the figures in the manuscript: SG, JK. All authors read and approved the final manuscript.

Acknowledgements

The authors thank Dr. Wen-Yue Xu from Department of Pathogenic biology, Third Military Medical University, Chongqing China, who provided us the mosquito. We also thank Mr. Hongxin Ou for his technical support. We further extend our thanks to Dr. Sarala K Subbarao, formerly Director National Institute of Malaria Research (Delhi, India) for her help in revising the manuscript.

References:

1. Gholizadeh S, Karimi NN, Zaker S, Djadid NN. The Role of Molecular Techniques on Malaria Control and Elimination Programs in Iran: A Review Article. Iran J Parasitol. 2018; 13(2);161-171.
2. Djadid ND, Gholizadeh S, Aghajari M, Zehi AH, Raeisi A, Zakeri S. Genetic analysis of rDNA-ITS2 and RAPD loci in field populations of the malaria vector, *Anopheles stephensi* (Diptera: Culicidae): implications for the control program in Iran. Acta Trop. 2006; 97:65–74.
3. Subbarao SK, Vasantha K, Adak T, Sharma VP, Curtis CF. Egg float ridge number in *Anopheles stephensi*: ecological variation and genetic analysis. Med Vet Entomol. 1987;1(3): 265-271.

4. Subbarao S K 1998 *Anopheles species complexes in South East Asia region* (Technical publication No. 18) (New Delhi:World Health Organization, South East Asia Regional Office).
5. World Health Organization. Regional Office for South-East Asia. (2007). New Year Address to Staff at SEARO, 31 December 2007, WHO/SEARO, New Delhi. WHO Regional Office for South-East Asia. https://apps.who.int/iris/handle/10665/126423.
6. Oshaghi MA, Yaghoobi F, Vatandoost H,Abaei MR, Akbarzadeh K. *Anopheles stephensi* biological forms: Geographical Distribution and Malaria Transmission in Malarious regions of Iran. Pak Bio Scie. 2006b;9 (2):294-298.
7. Oshaghi MA, Yaghobi-Ershadi MR, Shemshad K, Pedram M, Amani H. The Anopheles superpictus complex: introduction of a new malaria vector complex in Iran. Bull Soc Pathol Exot. 2008;101:429–34.
8. Gholizadeh S, Djadid ND, Nouroozi B, Bekmohammadi M. Molecular phylogenetic analysis of Anopheles and Cellia subgenus anophelines (Diptera: Culicidae) in temperate and tropical regions of Iran. Acta Trop. 2013b;126:63–74.
9. Sinka M, Bangs MJ, Manguin S, Chareonviriyaphap T, Patil AP, Temperley WH, Gething PW. The dominant Anopheles vectors of human malaria in the Asia-Pacific region: occurrence data, distribution maps and bionomic précis. Paras and Vec. 2011; 4:89.
10. World Health Organization (WHO). World Malaria Report 2018 and 2020. World Health Organization; 2018.
11. Dash AP, Adak T, Raghavendra K, Singh OP. The biology and control of malaria vectors in India. 2007; Curr Sci: 92, 1571–1578.
12. Faulde MK, Rueda LM, Khaireh BA. First record of the Asian malaria vector *Anopheles stephensi* and its possible role in the resurgence of malaria in Djibouti, Horn of Africa. Acta Trop. 2014;139:39–43.

13. Vipin, Dube M, Gakhar SK. Genetic differentiation between three ecological variants (‘type’, ‘mysorensis’ and ‘intermediate’) of malaria vector *Anopheles stephensi* (Diptera: Culicidae). Insect Science. 2010; 17: 335–343.

14. Basseri HR, Mohamadzadeh Hajipirloo H, Mohammadi Bavani M, Whitten MMA. Comparative Susceptibility of Different Biological Forms of *Anopheles stephensi* to *Plasmodium berghei* ANKA Strain. PLoS ONE. 2013; 8(9): e75413.

15. Firooziyan S, Djadid ND, Gholizadeh S. Speculation on the possibility for introducing *Anopheles stephensi* as a species complex: preliminary evidence based on odorant binding protein 1 intron I sequence. Malar J. 2018; 17:366.

16. Gholizadeh S, Firooziyan S, Ladonni H, Hajipirloo HM, Djadid ND, Hosseini A, Raz A. The *Anopheles stephensi* odorant binding protein 1 (*AnsteObp1*) gene: A new molecular marker for biological forms diagnosis. Acta Tropica. 2015; 146; 101–113.

17. Mishra S, Sharma G, Das MK, Pande V, Singh OP. Intragenomic sequence variations in the second internal transcribed spacer (ITS2) ribosomal DNA of the malaria vector *Anopheles stephensi*. PLoS ONE. 2021; 16(6): e0253173.https://doi.org/10.1371/journal.pone.0253173.

18. Alam MT, Bora H, Das MK, Sharma YD. The type and mysorensis forms of the *Anopheles stephensi* (Diptera: Culicidae) in India exhibit identical ribosomal DNA ITS2 and domain-3 sequences. Parasitol Res. 2008; 103:75–80.

19. Zhang J, Zhang S, Wang Y, Xu W, Zhang J, et al. Modulation of *Anopheles stephensi* Gene Expression by Nitroquine, an Antimalarial Drug against *Plasmodium yoelii* Infection in the Mosquito. PLoS ONE. 2014; 9(2).

20. Khan I, Farid A, Zeb A. Development of inexpensive and globally available larval diet for rearing *Anopheles stephensi* (Diptera: Culicidae) mosquitoes. Para and Vec. 2013; 6:90.
21. Larkin MA, Blackshields G, Brown NP, Chenna R, McGettigan PA, McWilliam H, Valentin F. et al. ClustalW and ClustalX version 2. Bioinformatics. 2007; 23 (21), 2947–2948.
22. Tamura K, Peterson D, Peterson N, Stecher G, Nei M, Kumar S. MEGA5: molecular evolutionary genetics analysis using maximum likelihood, evolutionary distance, and maximum parsimony methods. Mol. Biol. Evol. 2011; 28 (10): 2731–2739.
23. Sweet WC and Rao BA. Races of *A. stephensi* Liston. Indian Med Gaz. 1937;72:665–74.
24. ZouYL, HaoYJ, XuWY, DingYR, ZhouY, He QY, Chen B. NCBI direct submission (KT899888). 2015.
25. Stone AK, Knight L and Starcke H. (1959). A synoptic catalog of mosquito of the world (Diptera, culicidea). Thomas Say Foundation, Entomological society of America, Washington.
26. Puri IM (1949). Anophelines of the Oriental region. Malariology (ed. M.F. Boyd), pp, 483-505. W.B. Saunders, Philadelphia.
27. Rutledge LC, Ward RA, Bickley WE. Experimental hybridization of geographic strains of *Anopheles stephensi* (Diptera: Culicidae). Ann Entomol Soc Am. 1970; 63:1024–30.
28. Sweet WC, Rao BA, Subba Rao AM. Cross breeding of Anopheles stephensi type and Anopheles stephensi var. mysorensis. J Malar Inst Ind. 1938; 1:149–154
29. Anyanwu GI, Davies DH, Molyneux DH, Phillips A. Variation in cuticular hydrocarbons among strains of *Anopheles stephensi* Liston possibly related to prior insecticide exposure. Ann Trop Med Parasitol. 1997; 91: 649–659.
30. Coluzzi M, Di Deco M, Cancrini G. Chromosomal inversions in A*nopheles stephensi*. Parassitologia. 1973;15:129–36.
31. Suguna SG. The genetics of three larval mutants in *Anopheles stephensi*. Indian J Med Res. 1981; 73 (Suppl):120–3.

32. Mahmood F, Sakai RK. Inversion polymorphisms in natural populations of Anopheles stephensi. Can J Genet Cytol. 1984; 26:538–546.
33. Subbarao SK (1996). Genetics of malaria vectors. Proc. Nat Acad. India 66 (B). SPL. Issue: 51-76.
34. Suleman M. Intraspecific variation in the reproductive capacity of *Anopheles stephensi* (Diptera: Culicidae). J Med Entomol. 1990; 27:819–28.
35. World Mosquito Program. (2021) https://www.world mosquitoprogram.org/
36. Wang G, Li C, Zheng W, Song F, Guo X, Wu Z, Luo P, Yang Y, He L, Zhao T. An evaluation of the suitability of COI and COII gene variation for reconstructing the phylogeny of, and identifying cryptic species in, anopheline mosquitoes (Diptera Culicidae). Mitochondrial DNA Part A. 2016; 28 (5);769-777. https://doi.org/10.1080/24701394.2016.1186665
37. Surendran NS, Sivabalakrishnan K, Gajapathy K, Arthiyan S, Jayadas TTP, Karvannan K, Raveendran S, et al. Genotype and biotype of invasive *Anopheles stephensi* in Mannar Island of Sri Lanka. Para and Vec. 2018; 11:3;1-7.
38. Nagpal BN, Srivastava A, Kalra NL, Subbarao SK. Spiracular indices in Anopheles stephensi: a taxonomic tool to identify ecological variants. J Med Entomol. 2003;40(6):747–9.
39. Chavshin AR, Oshaghi MA, Vatandoost H, Hanafi-Bojd AA, Raeisi A, Nikpoor F. Molecular characterization, biological forms and sporozoite rate of *Anopheles stephensi* in southern Iran. Asian Pac J Trop Biomed. 2014; 4(1): 47-51.
40. Carter TE, Yared S, Gebresilassie A, Bonnell V, Damodaran L, Lopez K, Ibrahim M, Mohammed S, Janies D. First detection of *Anopheles stephensi* Liston, 1901 (Diptera: Culicidae) in Ethiopia using molecular and morphological approaches. Acta Tropica. 2018; S0001-706X(18)30561-8.
41. Rao, B. A., W. C. Sweet, and A. M. Subbarao. 1938. Ova measurements of *A. stephensi* type and *A. stephensi var.mysorensis*. J. Malar. Inst. India. 1: 261Ð266.

Table 1: Primers detail and their respective thermal profiles in PCR amplification

Primer Name	Sequence 5-3	Size (bp)	Thermal Profile	Reference
COIF	TTGATTTTTTGGTCATCCAGAAGT	877	DT: 94 °C (4 min), 94 °C(1m),<br>AT: 55 °C (1m)<br>ET: 72 °C (2m), 72 °C for (7m). 32 cycles	Chavshin et al., 2014 [39]
COIR	TAGAGCTTAAATTCATTGCACTAATC			
COII F	ATGGCA ACATGAGCAAATT	640		
COII R	CCACCCTTTCTGAACATTGACC			
ITS2 5.8s F	ATCACTCGGCTCGTGGATCG	650	DT: 95°C (2m) 95°C (30s), AT: 50°C (30s), ET: 72°C (1m), 72°C (5m). 30 cycles	Carter et al., 2018; [40] Djadid et al., 2006 [2]
ITS2 28s R	ATGCTTAAATTTAGGGGGTAGTC			
ANSTEOBP1 F	CGTAGGTGGAATATAGGTGG	900	DT: 95◦C (5m), 95◦C (1m) AT: 60◦C (1.20m), ET: 72◦C for 1.20m and further 10 m. 30 cycles	Gholizadeh et al., 2018 [1]
ANSTEOBP1 R	TCGGCGTAACCATATTTGC			

F: Forward, **R**: Reverse, **DT**: Denaturation Temperature, **AT**: Annealing Temperature,

ET: Extension Temperature. **m**: minute, **s**: second

Figure Legend

Figure 1. Scanning electron micrograph of *Anopheles stephensi 'mysorensis'* form egg: (a) Ventral aspect showing deck area; (b) Lateral aspect showing floats and ribs;(c) Lateral aspect.

Figure 2. Phylogeny of COI sequence from lab strain. Bootstrap values >70 shown at nodes. Nodes without numbers had a value <70. Final ML Optimization Likelihood: -1252.592081.

Figure 3. Phylogeny of COII sequence from lab strain. Bootstrap values>70 shown at nodes. Nodes without numbers had a value <70. Final ML Optimization Likelihood: -1252.592081.

Figure 4. Phylogeny of rDNA-ITS2 sequences from lab strain. Bootstrap values >70 shown at nodes. Nodes without numbers had a value <70. Final ML Optimization Likelihood: -1104.708501.

Figure 5. Multiple sequence alignment of *AnsteObp1* partial sequence of lab strain with three known biological forms of *An. stephensi*.

Figure 6. Phylogenetic three constructed based on *AnsteObp1* intron I region of *An. stephensi* sequences from Lab strain China and representative sequences from GenBank.

Supplementary Figures

Figure 1. Multiple sequence analysis of COI sequence from lab strain. Bootstrap values >70 shown at nodes.

Figure 2. Multiples sequence analysis of COII sequence from lab strain. Bootstrap values >70 shown at nodes.

Figure 3. Multiples sequence analysis of rDNA-ITS2 lab strain. Bootstrap values >70 shown at nodes.

Figure 1

Click here to access/download;Figure;Figure 1.tiff

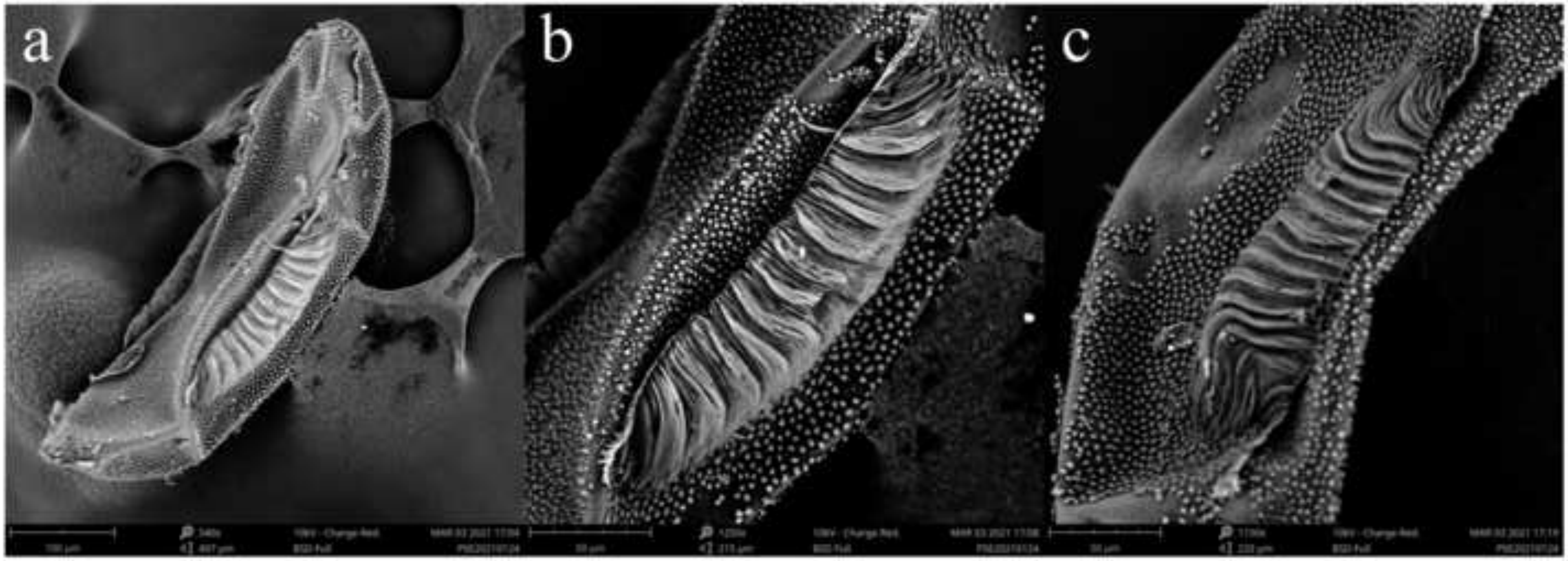

Click here to access/download;Figure;Figure 2.docx

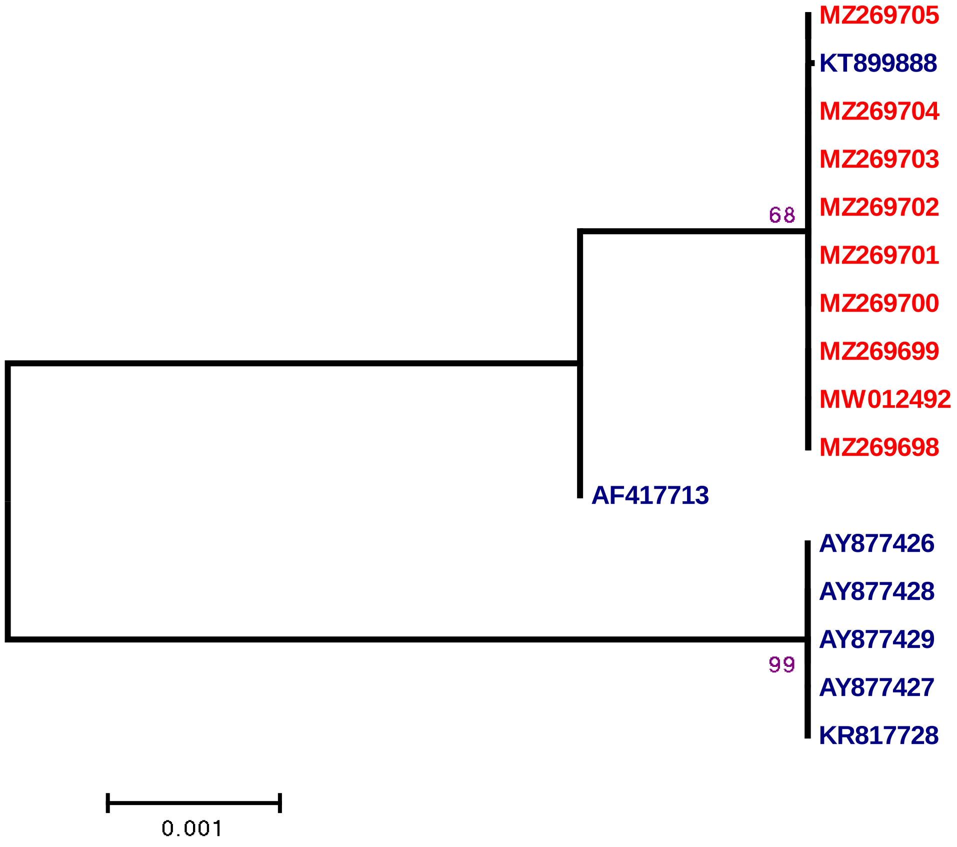

Figure 3

Click here to access/download;Figure;Figure 3.docx

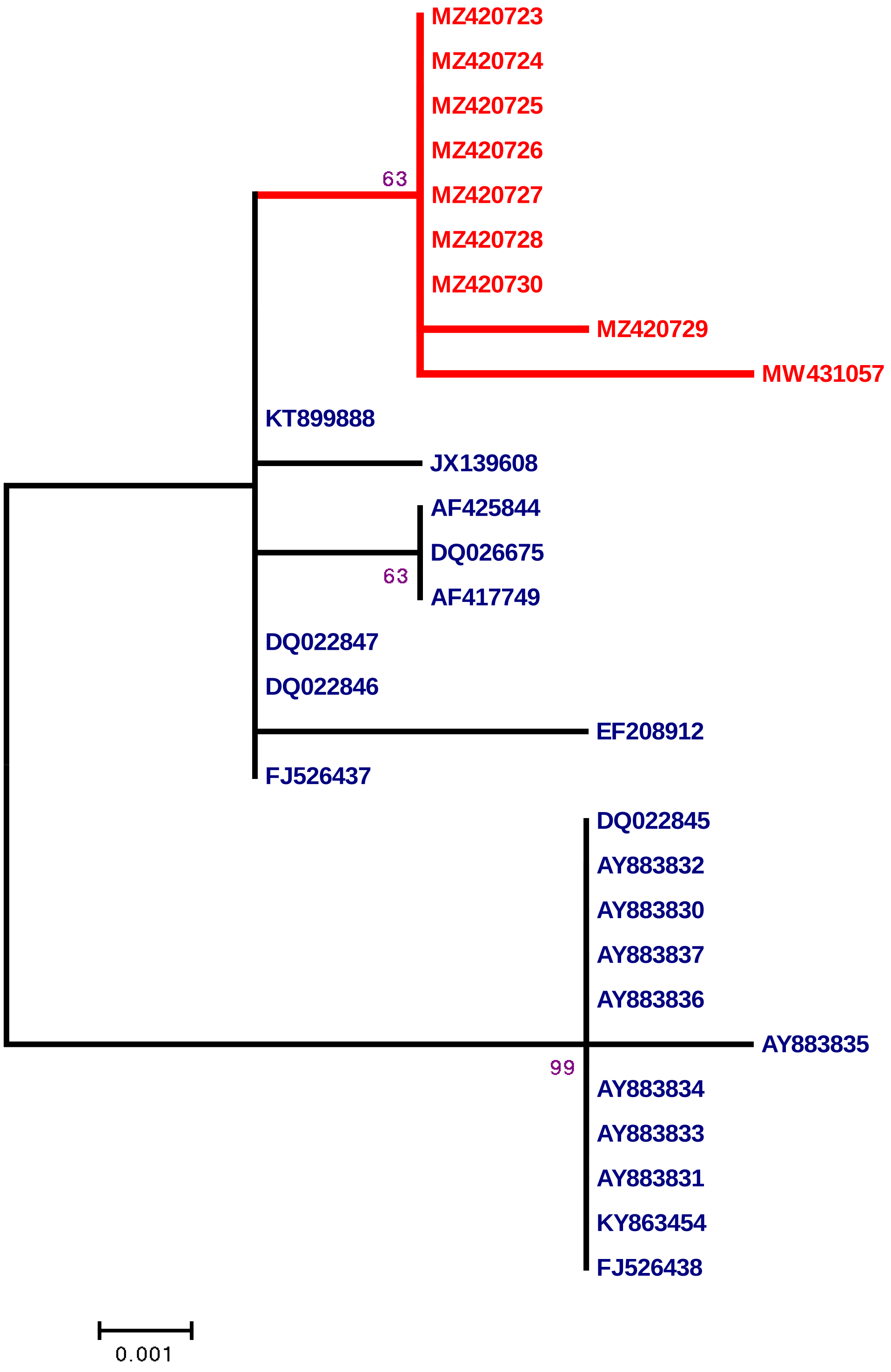

Figure 4

Click here to access/download;Figure;Figure 4.docx

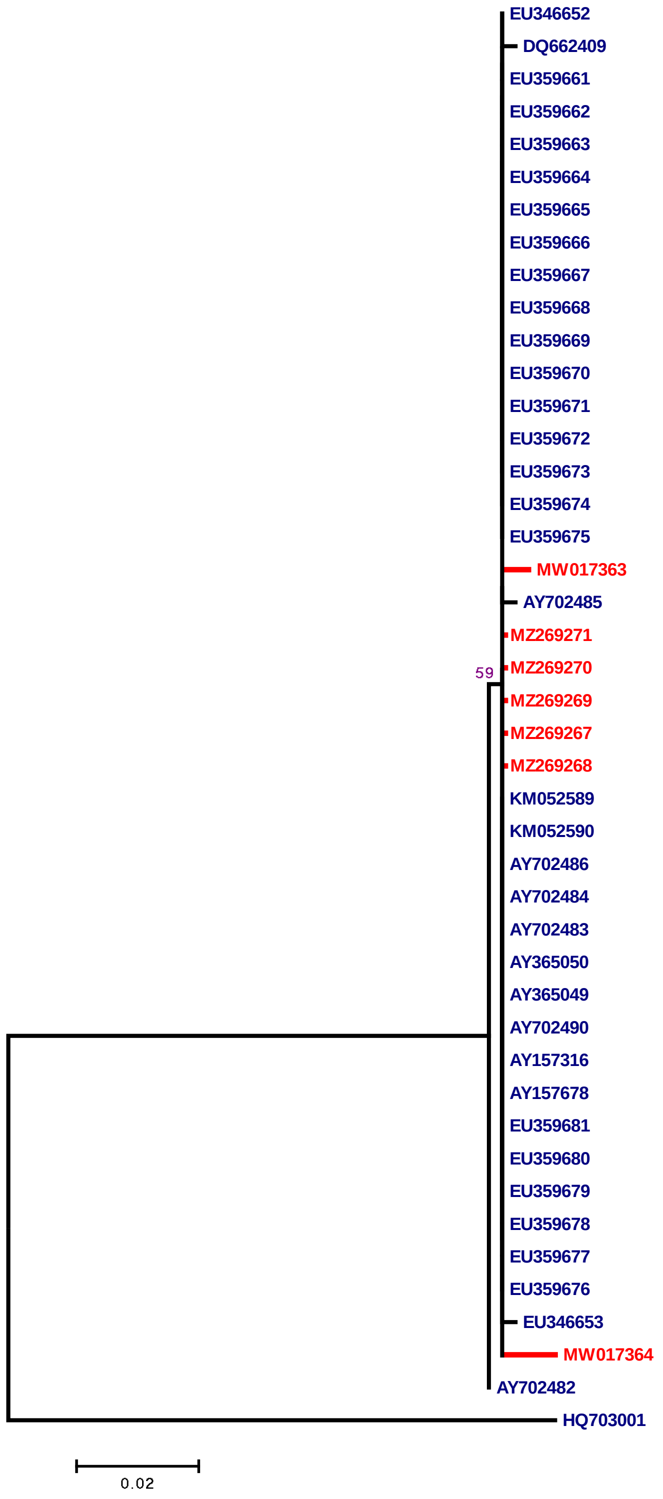

Figure 5

Click here to access/download;Figure;Figure 5.docx

```
#KJ557455_An._stephensi_C_(mysorensis)      GTGAGCTTGG GTGTCTTCTG GATATTGCTC TAATGTGTTT TTACTCTACT TGCTTTTGAC  [ 60]
#KJ557452_An._stephensi_B_(intermediate)    .....T.... .......... .......T.. .......... .CTG....TA A.T.......  [ 60]
#KJ557463_An._stephensi_A_(type)            .......... .......... .......T.. .......... .CTG....TA A.T...AA..  [ 60]
#MW013512                                   .......... .......... .......... .......... .......... ..........  [ 60]
#MW013513                                   .......... .......... .......... .......... .......... ..........  [ 60]
#MW013514                                   .......... .......... .......... .......... .......... ..........  [ 60]
#MW013515                                   .......... .......... .......... .......... .......... ..........  [ 60]
#MW013516                                   .......... .......... .......... .......... .......... ..........  [ 60]
#MW013517                                   .......... .......... .......... .......... .......... ..........  [ 60]
#MW013518                                   .......... .......... .......... .......... .......... ..........  [ 60]
#MW013519                                   .......... .......... .......... .......... .......... ..........  [ 60]
#MW013520                                   .......... .......... .......... .......... .......... ..........  [ 60]
#MZ420719                                   .......... .......... .......... .......... .......... ..........  [ 60]
#MZ420720                                   .......... .......... .......... .......... .......... ..........  [ 60]
#MZ420721                                   .......... .......... .......... .......... .......... ..........  [ 60]
#MZ420722                                   .......... .......... .......... .......... .......... ..........  [ 60]

#KJ557455_An._stephensi_C_(mysorensis)      AGAAATCTGA ATTCTGAATG TTAAATATAA TCTCCTGTCA TGCAATGTCA TCACTTTCCA  [120]
#KJ557452_An._stephensi_B_(intermediate)    .C.......G .C....C..C C....G.... .T..-----. .......... ..........  [120]
#KJ557463_An._stephensi_A_(type)            .C.......G .C....C..C C....G.... .G........ .......... ..........  [120]
#MW013512                                   .......... .......... .......... .......... .......... ..........  [120]
#MW013513                                   .......... .......... .......... .......... .......... ..........  [120]
#MW013514                                   .......... .......... .......... .......... .......... ..........  [120]
#MW013515                                   .......... .......... .......... .......... .......... ..........  [120]
#MW013516                                   .......... .......... .......... .......... .......... ..........  [120]
#MW013517                                   .......... .......... .......... .......... .......... ..........  [120]
#MW013518                                   .......... .......... .......... .......... .......... ..........  [120]
#MW013519                                   .......... .......... .......... .......... .......... ..........  [120]
#MW013520                                   .......... .......... .......... .......... .......... ..........  [120]
#MZ420719                                   .......... .......... .......... .......... .......... ..........  [120]
#MZ420720                                   .......... .......... .......... .......... .......... ..........  [120]
#MZ420721                                   .......... .......... .......... .......... .......... ..........  [120]
#MZ420722                                   .......... .......... .......... .......... .......... ..........  [120]
```

Click here to access/download;Figure;Figure 6.docx

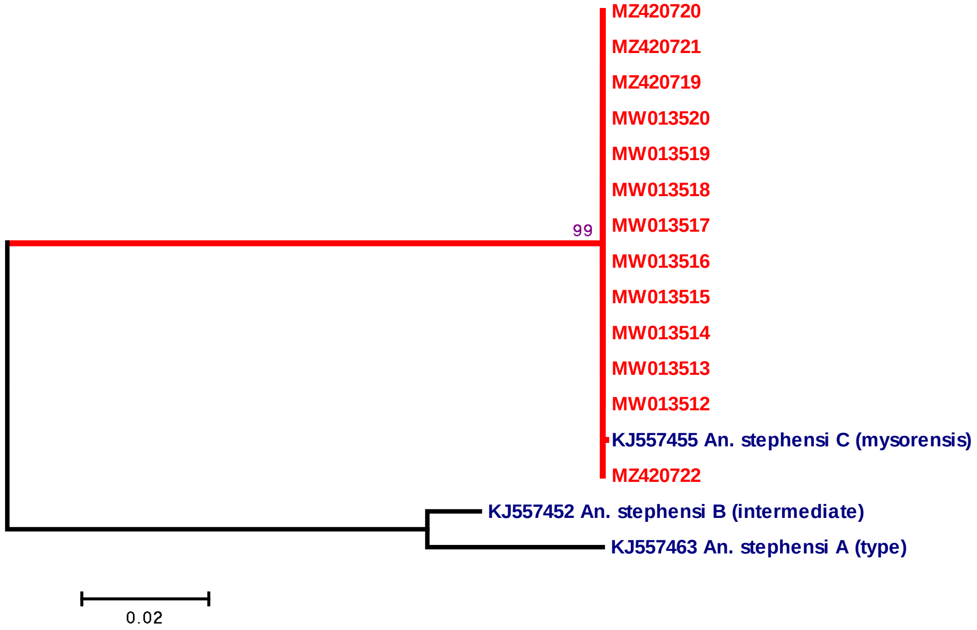

Supplementary figure 1

Click here to access/download
Supporting Information
Suppl figure 1 .docx

Supplementary figure 3

Click here to access/download
Supporting Information
Suppl figure 3.docx

Click here to access/download
Supporting Information
Suppl Table.docx

Supplementary figure 2

Click here to access/download
Supporting Information
Suppl figure 2.docx
