# Supplementary material for: Identification of a biological form in the Anopheles stephensi laboratory colony using the odorant-binding protein 1 intron I sequence
Source: PLoS One. 2022 Feb 22;17(2):e0263836. doi: 10.1371/journal.pone.0263836 (PMC8863247; doi:10.1371/journal.pone.0263836)
Supplement: Attachment — Submitted filename: Response to Reviewers.docx [file pone.0263836.s006.docx]

Dear Editor,

We have addressed all the points raised by the reviewers. The corrections and modifications in the manuscript have been made accordingly. Furthermore, after publication, the sequences of our manuscript will be publicly available (with working accession numbers) at NCBI.

**The responses to each reviewer's individual points are listed below.**

**Reviewer #1**:

Page 3: Authors claim "AnsteObp1 as a robust genetic marker for rapid and accurate discrimination (taxonomic identification) of the An. stephensi species complex".
Page 11: " Finally, our investigations, based on egg morphology and sequence analysis, endorse the use (independent) of the AnsteObp1 intron I sequence as a new molecular tool for quick and reliable identification of all the three biological forms of An. stephensi."

First: this claim had been proposed before on 2015, so this is not a "new" molecular tool.

**Reply:** We have replaced the word ‘new’ with ‘suitable’.
Second: In this study only An. stephensi mysorensis lab strains were used. No wild anopheles and no type and intermediate forms were used.

**Reply:** We have replaced the ‘three biological forms of *An.stepehnsi*’ with ‘*An. stephensi* mysorensis’.
**Third:** how would you use this marker to separate Type and Intermediate forms from Mysorensis? The answer to this question is not clearly mentioned in the manuscript.

**Reply**: This question has already been addressed in the manuscript in the discussion, such as ‘This identification was attributed to the form specific/associated mutations (4-15%) within the intron region of *Anste*Obp1 between biological……… [16]’. Moreover, we have cited the relevant study by Gholizadeh et al. (2015) [16] and further details may be seen by readers.

Phylogenetic trees in Fig.2-3 -4: Comparison between sequences is not clearly mentioned in the phylogenetic tree by name/region.

**Reply**: Comparison between sequences is now clearly mentions the phylogenetic tree by name/region.

**Reviewer #2**: 
The title is not appropriate for this work as it does not introduce a new marker for species discrimination. I suggest changing the title to “Identification of a biological form in the *Anopheles stephensi* laboratory colony using the odorant-binding protein 1 intron I sequence.”

**Reply:** We have changed the title as suggested.
The authors should clearly state the limitations of their work.
First, the test was limited to a single laboratory colony. Second, the authors have not developed a rapid PCR-based test and identification of the biological form requires sequencing, which is labor- and time-consuming. Third, only a single (the last) SNP discriminate all three forms: “C” in mysorensis, “T” in intermediate, “G” in type. Other polymorphisms are shared between at least two forms.

**Reply:** The limitations have been included now.

The authors wrote that they counted for the number of ridges under stereomicroscope, however Figure 1 shows a scanning electron micrograph. Please, clarify and correct this, and also provide scale bars.

**Reply:** We counted the number of ridges under stereomicroscope according to the previously published protocols (common methods) [3, 16] but we used scanning electron microscopy to present a clearer image. Now, we have added this sentence “In addition, a scanning electron microscopy image was taken to clearly show the egg form” in the concerned section. Moreover, the scale bar values are added (mentioned) now in the figure legend.

**Please, correct the following:
Abstract:**
1. *An. stepehnsi* species complex - change to - The *An. stephensi* species complex

**Reply:** Corrected.

2. vector competence (malaria) and ecology - change to - vector competence to the malaria parasite and ecology.

**Reply:** Corrected.

3. To identify the species complex of our *An. stephensi* insectary colony - change to - To identify the members of the species complex in our An. stephensi insectary colony.

**Reply:** Corrected.

4. Eggs were collected from individual mosquito - change to - Eggs were collected from individual mosquitoes.

**Reply:** Corrected.

5. sequences in GenBank using MEGA vx. - change to - sequences in GenBank using MEGA 7.

**Reply:** Corrected.

6. An. stephensi sibling C - change to - An. stephensi sibling species C
**Reply:** Corrected.

**Main text**:
**1**. Despite efficient controlling strategies for malaria, An. stephensi is increasing in its geographic range (add reference).

**Reply:** reference added.

**2**. chromosome karyotypes [30,31,32,33], cytogenetic properties [30,31]. Recently, AnsteObp1 is reported as a new marker for identification of the Asian main malaria vector, An. stephensi (add reference)

**Reply:** Reference added.

**3**. amino acids sequences of AnsteObp1 among these members - change to - amino acids sequences of AnsteObp1 among these members.

**Reply:** Corrected.

**4**. Wolbachia-based, Gene drive etc. - change to - Wolbachia-based, gene drive, etc.

**Reply:** Corrected.

**5**. Others demonstrated variations in the reproductive capacity within these biological forms of An. stephensi [34]. - change to - Others demonstrated variations in the reproductive capacity within An. stephensi [34].

**Reply:** Corrected.

Table 1: Size of ANSTEOBP1 is 900 bp, but it should be 845 bp.

**Reply:** Corrected.
